# B cells modulate lung antiviral inflammatory responses via the neurotransmitter acetylcholine

**DOI:** 10.1038/s41590-025-02124-8

**Published:** 2025-04-22

**Authors:** Antonio Cembellin-Prieto, Zheng Luo, Heather Kulaga, Nicole Baumgarth

**Affiliations:** 1 Graduate Group in Immunology, University of California Davis, Davis, CA USA; 2 W. Harry Feinstone Department of Molecular Microbiology and Immunology, Lyme and Tickborne Diseases Research and Education Institute, Johns Hopkins Bloomberg School of Public Health, Johns Hopkins University, Baltimore, MD USA; 3 Department of Pathology, Microbiology and Immunology, School of Veterinary Medicine, University of California Davis, Davis, CA USA; 4 Department of Molecular and Comparative Pathobiology, School of Medicine, Johns Hopkins University, Baltimore, MD USA

**Keywords:** Innate immunity, Influenza virus, Acute inflammation, Adaptive immunity

## Abstract

The rapid onset of innate immune defenses is critical for early control of viral replication in an infected host and yet it can also lead to irreversible tissue damage, especially in the respiratory tract. Sensitive regulators must exist that modulate inflammation, while controlling the infection. In the present study, we identified acetylcholine (ACh)-producing B cells as such early regulators. B cells are the most prevalent ACh-producing leukocyte population in the respiratory tract demonstrated with choline acetyltransferase (ChAT)-green fluorescent protein (GFP) reporter mice, both before and after infection with influenza A virus. Mice lacking ChAT in B cells, disabling their ability to generate ACh (ChatBKO), but not those lacking ChAT in T cells, significantly, selectively and directly suppressed α7-nicotinic-ACh receptor-expressing interstitial, but not alveolar, macrophage activation and their ability to secrete tumor necrosis factor (TNF), while better controlling virus replication at 1 d postinfection. Conversely, TNF blockade via monoclonal antibody treatment increased viral loads at that time. By day 10 of infection, ChatBKO mice showed increased local and systemic inflammation and reduced signs of lung epithelial repair despite similar viral loads and viral clearance. Thus, B cells are key participants of an immediate early regulatory cascade that controls lung tissue damage after viral infection, shifting the balance toward reduced inflammation at the cost of enhanced early viral replication.

## Abstract (web summary)

Cembellin-Prieto and colleagues find that B cells in the lung are capable of producing acetylcholine and that these regulate early macrophage activation and control influenza virus replication.

## Main

Respiratory tract infections can cause severe illnesses as a result of dysregulated inflammatory responses, including overshooting production of proinflammatory cytokines such as TNF. Interstitial lung macrophages play a particular role as elaborators of proinflammatory cytokines^1^. TNF production by macrophages has also been associated with increased viral control after influenza infection, highlighting the need to balance immune activation for viral clearance with that of control for overshooting inflammation^2–6^. There is an urgent clinical need to identify control mechanisms of pulmonary inflammation, especially those associated with viral infections, where poor prognoses are often associated with dysregulated inflammation^7–12^.

B cells are known foremost for antibody production, yet they can also present antigens and produce cytokines and metabolites. Granulocyte–macrophage colony-stimulating factor (GM-CSF) and interleukin (IL)-10 production by B cells were shown to regulate early immune responses, including in the lung^13–20^. Production of metabolites and the neurotransmitters γ-aminobutyric acid (GABA) and acetylcholine (ACh) are additional, but less well understood, effector functions of B cells^21–27^. ACh is of interest, because it is a neurotransmitter generated from choline and acetyl-coenzyme A via the action of choline acetyltransferase (ChAT)^28,29^, which functions as both a controller of autonomic body functions^30–34^ and an immunoregulator, functions that are increasingly being explored therapeutically^35–49^. ChAT-GFP reporter mice demonstrated ChAT expression by a variety of leukocytes^27,50–56^ with ACh-producing T cells identified as critical components of the cholinergic inflammatory pathway, regulating macrophage functions in the spleen^51,52^. However, most ChAT-expressing leukocytes are B cells^27^. In the present study, we identified these ChAT-expressing B cells as critical modulators of interstitial macrophage-induced lung inflammation and virus replication during influenza virus infection.

## TNF regulates viral loads and is a target of ACh

**Fig. 1:**
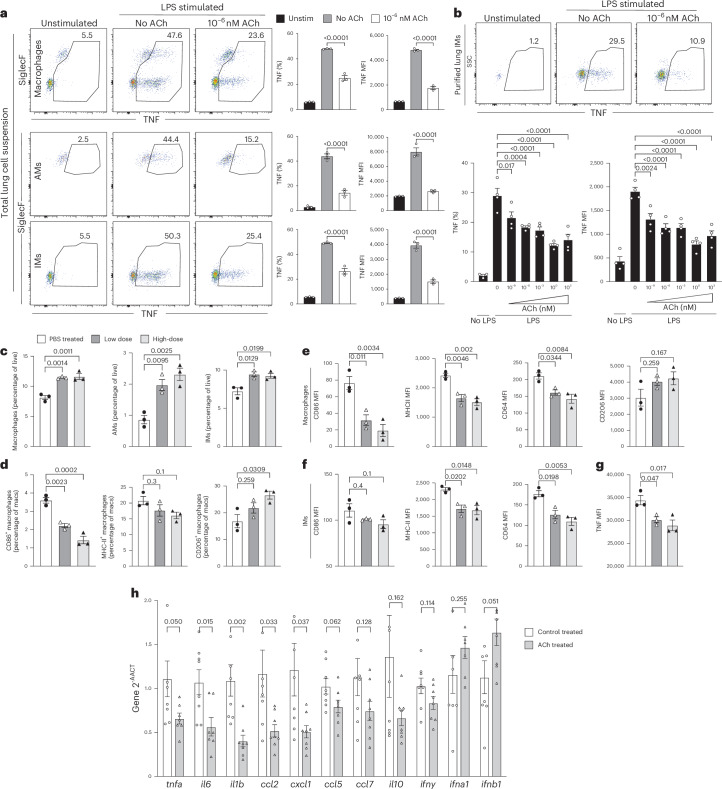
TNF is a target of ACh. **a**, Total lung single-cell suspensions from C57BL/6 mice (*n* = 2) cultured in the presence or absence of LPS and treated or not with ACh, in the presence of brefeldin A for 5 h at 37 °C. Representative flow cytometry plots (left) and frequencies of TNF-expressing lung total macrophages (right, top: CD19^−^, ThY1.2^−^, Ly6G^−^, Ly6C^−^ and F4/80^+^/CD64^+^), AMs (middle: further gated on CD11b^−^, CD11c^+^ and SiglecF^+^) or IMs (bottom: further gated on CD11b^+^, CD11c^−^ and SiglecF^−^) are plotted as the frequency of the previous gate (left) or expressed as median fluorescence intensity (MFI, right). **b**, Negatively enriched IMs from C57BL/6 mice (*n* = 5) cultured with indicated doses of ACh as in **a**. Representative flow cytometry plots (top) and frequencies of TNF-expressing IMs and TNF MFI (bottom) are shown. **c**–**f**, C57BL/6 mice treated i.n. with a combination of ACh and PB 12 h before, at the time of and 24 h after infection with 10 p.f.u. of A/PR8 (0 h), and lungs analyzed via flow cytometry at 1 d.p.i. **c**,**d**, Frequencies of lung parenchyma total macrophages (macs), AMs and IMs (percentage of live) (**c**) and frequencies of lung parenchyma macrophages expressing CD86, MHC-II and CD206, respectively (**d**). **e**,**f**, MFI of indicated markers in total macrophages (**e**) and IMs (**f**), gated as in **a**. **g**, MFI of TNF expression in IMs, ex vivo, restimulated with LPS. **h**, Relative gene expression of indicated genes in lung homogenates from control-treated and ACh-treated mice at 1 d.p.i. with influenza A/PR8 (*n* = 7). Panels **a** and **b** represent three independent experiments giving similar results and data contain *n* = 3 (**a**) and *n* = 4 (**b**) total replicates per group. Panels **d** and **h** represent two independent experiments with *n* = 3 mice each. In **a**–**h**, the bar graphs show the mean ± s.e.m., in **a**–**g**, one-way analysis of variance (ANOVA) was used and, in **h**, the two-tailed, unpaired Student’s *t*-test. Unstim, unstimulated. Source data

**Extended Data Fig. 1:**
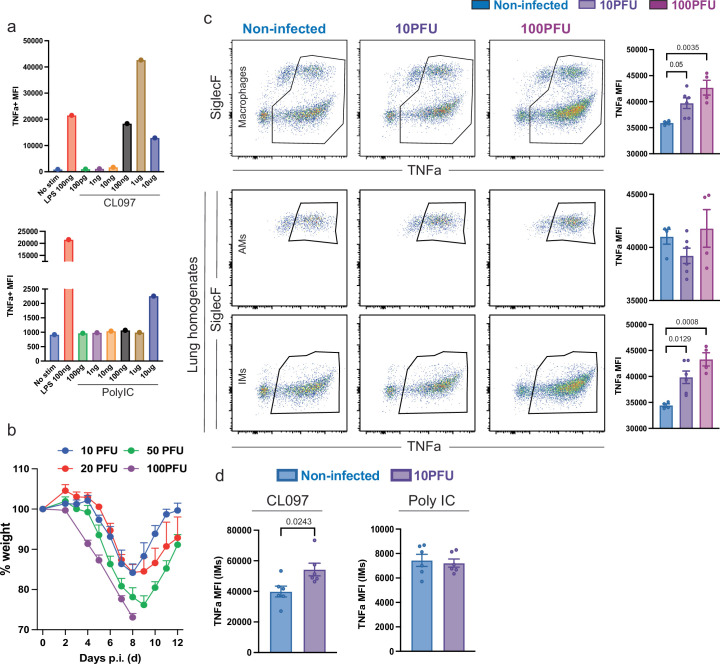
ACh inhibits pro-inflammatory cytokine production by lung interstitial macrophages *after influenza infection.* **a**) total lung single cell suspensions from C57BL/6 mice (n = 2) cultured in the presence or absence of depicted concentrations of stimuli: LPS, CL097 (top) and PolyIC (bottom). Shown are the MFI of TNFα-expression among IMs gated as in **c**). **b**) C57BL/6 mice (n = 4/group) were infected with indicated plaque-forming units (PFU) of influenza A/PR8 on d0 for 10-15 days. Shown is the mean percentage weight change + SD over the course of the infection compared to d0. **c**) C57BL/6 mice were infected with indicated doses of influenza A/PR8 i.n. (n = 4/non-infected and 10PFU group; n = 6/100PFU group) and lungs were analyzed by flow cytometry at 1 day post infection. Shown are representative flow cytometry plots (left) and MFI of TNFα-expression among lung total macrophages (right) gated on CD19-, Thy1.2-, Ly6G-, Ly6C-, F4/80 + /CD64+ (top), alveolar macrophages (AMs) further gated on CD11b-, CD11c + , SiglecF+ (middle), or interstitial macrophages (IMs) further gated on CD11b + , CD11c-, SiglecF- (bottom). **d**) C57BL/6 (n = 6/group) mice were infected as in **c**). MFI of TNFa-expression among IMs after ex-vivo restimulation with CL097 (left) or PolyIC (right). **a**) representative of two independent experiments **b**) n = 4/group mice pooled from 2 independent experiments. **c**) Results were pooled from 2 independent experiments. **d**) mice pooled from 2 independent experiments. Bar graphs show mean ± s.e.m. Symbols indicate results from an individual mouse. One-Way ANOVA. Source data

Interstitial macrophages (IMs) and alveolar macrophages (AMs) are critical cellular components of immune defenses to infections of the respiratory tract^1^. To assess the effects of ACh on lung inflammatory responses, it was applied to either total lung leukocytes or enriched lung-derived IMs in culture and TNF secretion was measured after short-term in vitro restimulation. Lipopolysaccharide (LPS) and toll-like receptor 7 (TLR7) agonist CL097 strongly induced TNF, whereas the TLR3 agonist poly(inosinic:polycytidylic acid) (poly(I:C)) induced TNF only weakly (Extended Data Fig. 1a). Therefore, LPS was used in all subsequent experiments, unless otherwise specified. ACh reduced frequencies of TNF-secreting, CD64^+^/F4/80^+^ total macrophages, as well as CD11b^−^CD11c^+^SiglecF^+^ lung homogenate AMs and CD11b^+^CD11c^−^SiglecF^−^ IMs by >50% (Fig. 1a). ACh also reduced TNF production by enriched lung IMs (Fig. 1b). Next, ACh and its stabilizer, the acetylcholinesterase inhibitor pyridostigmine bromide (PB), were applied intranasally (i.n.) to mice 12 h earlier, at the time of, and 24 h after, intranasal infection with influenza A/Puerto Rico/8/34 (A/PR8) (Fig. 1c). In this sublethal infection model, virus replication peaks by 2–3 d postinfection (d.p.i.) and virus-dose-dependent weight loss peaks around 7–9 d d.p.i. (Extended Data Fig. 1b). Application of ACh enhanced AM numbers in the lung parenchyma (Fig. 1d), suggesting their migration from the airways into the tissue. Both frequencies (Fig. 1e) and expression levels (Fig. 1f) of CD86 and of major histocompatibilty complex (MHC)-II on lung macrophages decreased, whereas surface expression levels of the activation inhibitor CD206^+^ increased (Fig. 1e). IMs showed dose-dependent decreased CD86, MHC-II and CD64 expression (Fig. 1g) and TNF production was significantly reduced (Fig. 1h), as were messenger RNA expression levels of proinflammatory genes *tnfa*, *il6* and *il1b*, and chemokines *ccl2*, *cxcl1*, *ccl5* and ccl7. Although *Il10* and *Ifng* transcript levels were unaffected, *Ifnb* levels increased (Fig. 1i). Thus, ACh can modulate lung AM and IM functions.

**Fig. 2:**
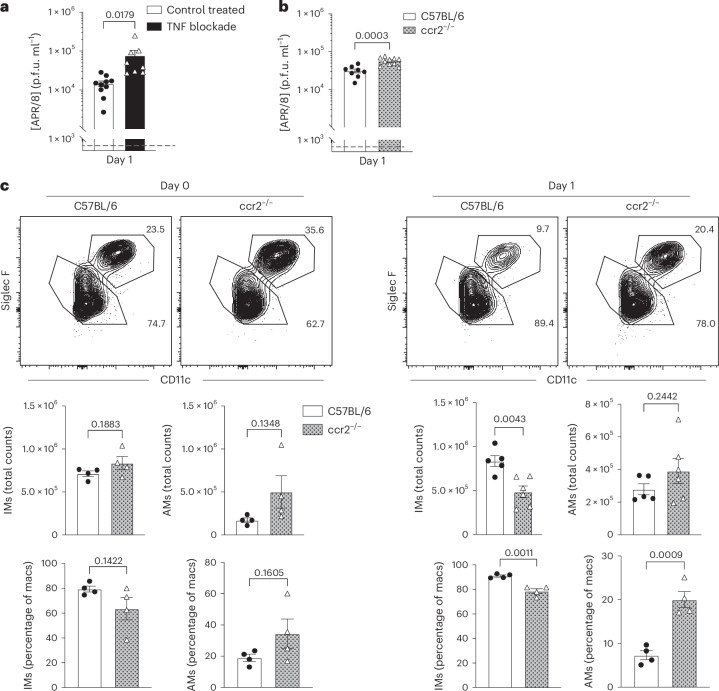
Inhibition of lung influenza virus replication by TNF and myeloid cell migration. C57BL/6 mice were treated with a blocking anti-TNF antibody 12 h before, at the time of and 6 h postinfection with 10 p.f.u. of APR/8. **a**,**b**, Influenza A/PR8 viral loads (p.f.u. ml^−1^) at 1 d.p.i. in lung homogenates from control-treated (*n* = 10) and anti-TNF-treated (*n* = 8) mice, pooled from two independent experiments (**a**) and C57BL/6 (*n* = 8) and *ccr2*^−/−^ (*n* = 10) mice pooled from two independent experiments (**b**). **c**, Top, representative flow plots of IMs (CD19^−^, ThY1.2^−^, Ly6G^−^, Ly6C^−^ and F4/80^+^/CD64^+^, further gated on CD11c^−^ and SiglecF^−^) and AMs (further gated on CD11c^+^ and SiglecF^+^). Middle, total count of lung parenchyma IMs (left) and AMs (right). Bottom, frequencies of IMs and AMs (percentage of previous gated macrophages) (*n* = 4 (left) and *n* = 5 (right) per group). The bar graphs show the mean ± s.e.m. In **a**–**c**, the two-tailed, unpaired Student’s *t*-test was used. Source data

TNF and nuclear factor κB (NF-κB) signaling pathways are known regulators of early innate responses to influenza infection^57^ and influenza infection results in enhanced TNF production by lung IMs but not AMs, (Extended Data Fig. 1c,d), suggesting that IMs are a major source of influenza-induced TNF. TNF blockade (Fig. 2a) strongly increased lung viral loads compared with sham-treated C57BL/6 controls at 1 d.p.i. (Fig. 2b), indicating a critical role for elaboration of TNF in early viral control. Mice deficient in *ccr2* lack inflammation-induced migration of macrophages and other innate immune cells into inflamed tissues. Their lung viral loads more than doubled by 1 d.p.i. compared with wild-type C57BL/6 mice at 1 d.p.i. but not before infection (Fig. 2c), which correlated with significant reductions in total lung IMs by both frequency and total numbers at 1 d.p.i. Lung AMs were not significantly altered by the lack of CCR2 (Fig. 2d). The more modest effects of *ccr2* deficiency on viral loads compared with that seen after TNF blockade is explained by the unaltered presence of tissue-resident IMs before infection (Fig. 2d). Thus, tissue-resident and infection-induced lung IMs control lung viral loads, at least in part by secreting TNF, which can be suppressed by ACh.

## B cells are the dominant ChAT expressers in the lung

**Fig. 3:**
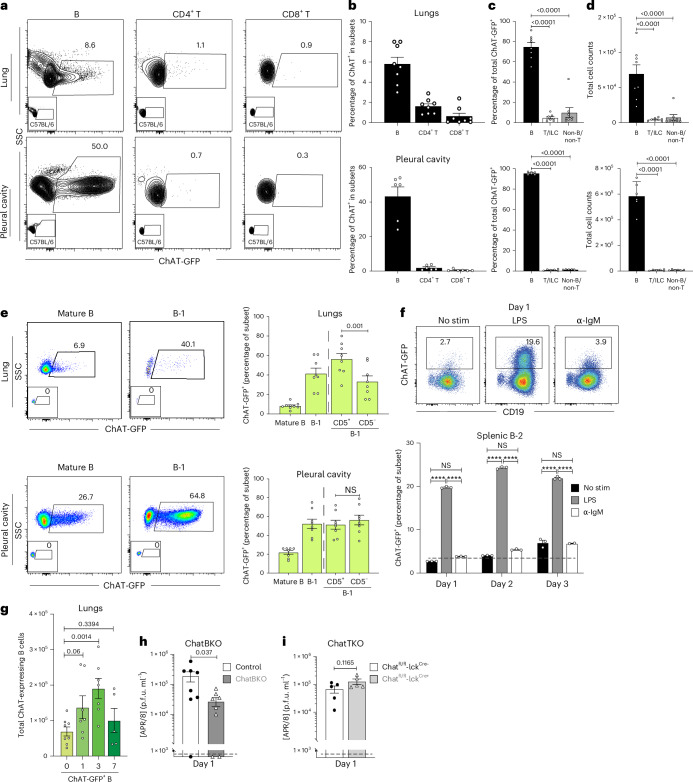
B cells are the major ChAT-expressing leukocytes regulating TNF production by IMs. **a**, Representative flow plots identifying ChAT-GFP^+^ B, CD4^+^ and CD8^+^ T cells from the lungs (top) and pleural cavity (bottom) of ChAT-GFP reporter mice. The small inserts show the same populations from C57BL/6 mice. **b**, ChAT-GFP^+^ frequencies among CD19^+^ B cells, CD3^+^CD4^+^ and CD3^+^CD8^+^ T cells in the lungs (top) and pleural cavity (bottom). **c**,**d**, Frequencies (**c**) and total cell counts (**d**) of ChAT-GFP^+^ cells that are B (CD19^+^), T/ILC (CD3^+^ or CD90.2^+^) or non-B/non-T (CD19^−^CD3^−^ and CD90.2^−^) in the lungs and pleural cavity. **e**, Representative flow plots (left) and frequencies (right) of ChAT-GFP^+^ B cell subsets in the lungs (top) and pleural cavity (bottom). **f**, Representative flow cytometry plots (top) and frequencies (bottom) of ChAT-GFP expression among magnetically enriched, total splenic B-2 cells (CD19^+^, CD23^+^, CD43^−^, CD5^−^ and CD9^−^) from ChAT-GFP reporter mice cultured in the presence or absence of LPS, anti-IgM (Fabʹ)_2_ or both, for the indicated times. **g**, Total ChAT-GFP B cell counts in the lungs of reporter mice infected for the indicated days postinfection with influenza A/PR8. **h**,**i**, Influenza A/PR8 viral loads at 1 d.p.i. from mb-1Cre^−/−^ChAT^fl/fl^ (control) and mb-1Cre^+/−^ChAT^fl/fl^ mice (ChatBKO) (*n* = 7–9) (**h**) and Chat^fl/fl^-lck-Cre^−/−^ (control) and Chat^fl/fl^-lck-Cre^+/−^ (ChatTKO) mice (*n* = 5) (**i**). In a–e, *n* = 8 (lung) or *n* = 6 (pleural cavity) mice were pooled from three independent experiments. In **f**, the data contain *n* = 3 replicates per group. In **g**, *n* = 8 mice, pooled from three independent experiments, in **h**, *n* = 8 mice, pooled from two independent experiments and, in **i**, *n* = 5 mice pooled from two independent experiments. In **a**–**i**, the bar graphs show the mean ± s.e.m. and, in **b**–**d**, **f** and **g**, one-way ANOVA and, in **e**, **h** and **i**, two-tailed, unpaired Student’s *t*-test were used. NS, not significant. Source data

**Extended Data Fig. 2:**
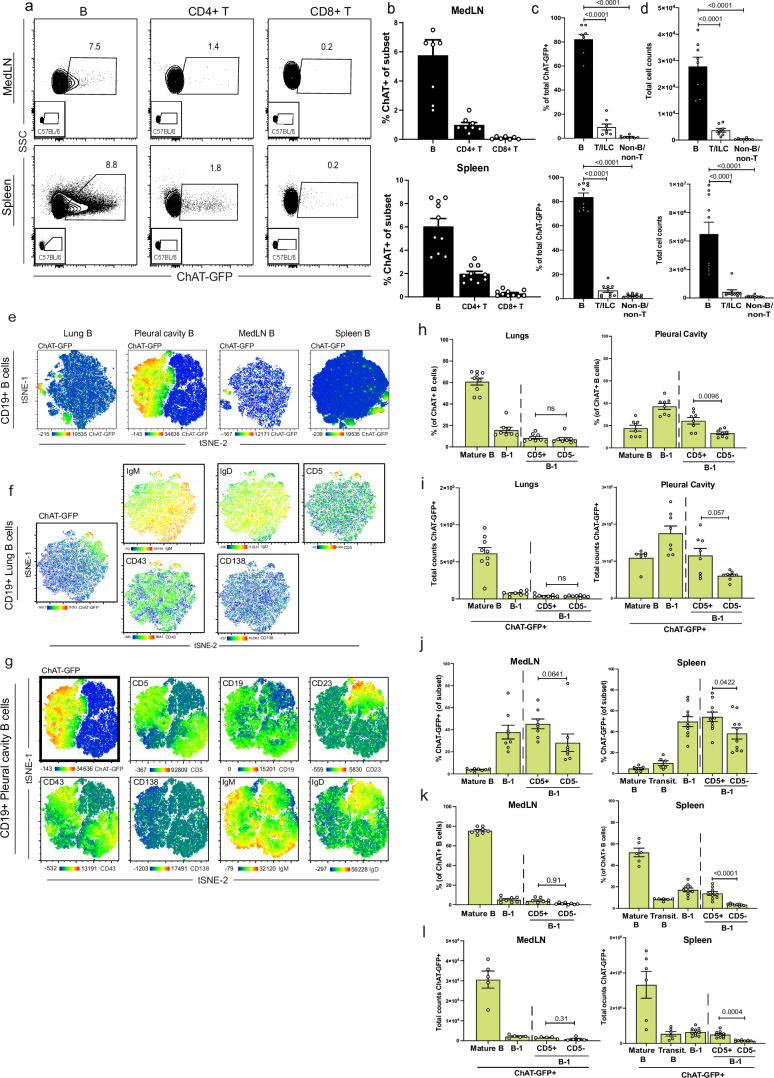
B cells are the major ChAT-expressing leukocytes at steady state. **a**) representative flow cytometry plots for the identification of ChAT-GFP + B, CD4+ and CD8 + T cells from mediastinal lymph node (MedLN) (top) and spleen (bottom) of GFP-ChAT reporter mice. Small inserts show same populations from non-GFP expressing C57BL/6 mice. **b**) ChAT-GFP+ frequencies among CD19 + B cells, CD3 + CD4+ and CD3 + CD8 + T cells in the MedLN (top) and spleen (bottom). **c**) frequencies of ChAT-GFP+ cells that are either B (CD19 + ), T/ILC (CD3+ or CD90.2 + ) or Non-B/Non-T (CD19- CD3-, CD90.2-). **d**) cell counts of ChAT-GFP + B, T/ILC and Non-B/Non-T cells in the MedLN (top) and spleen (bottom). **e**) tSNE plots with heat map indicating ChAT-GFP expression of B cells (CD19 + , Thy1.2-) from indicated tissues (lungs, pleural cavity, MedLN and spleen). **f**) flow cytometry tSNE plots of lung B cells (CD19 + , Thy1.2-) with heat map quantifying expression of indicated surface markers. **g**) flow cytometry tSNE plots of pleural cavity B cells (CD19 + , Thy1.2-) with heat map quantifying expression of indicated surface markers **h**) ChAT-GFP+ frequencies of B cell subsets (gating strategy, Extended Data Fig. 3) among total ChAT-GFP + B cells (CD19 + , Thy1.2-) in the lung and pleural cavity. **i**) cell counts of ChAT-GFP + B cell subsets in the lungs and pleural cavity. **j**) frequencies of ChAT-GFP+ cells among B cell subsets in the MedLN and spleen. **k**) frequency of ChAT-GFP+ frequencies of B cell subsets among total ChAT-GFP + B cells (CD19 + , Thy1.2-) in the MedLN and spleen. **l**) cell counts of ChAT-GFP + B cell subsets in the MedLN and spleen. **a**-**d**) n = 8/MedLN and n = 10/spleen mice pooled from 3 independent experiments. **e**-**g**) representative of 2 independent experiments with n = 3 mice per tissue. **h**-**l**) n = 8/lung and Pleural cavity; n = 8/MedLN and n = 10/spleen mice pooled from 3 independent experiments. Bar graphs indicate mean ± s.e.m. Symbols indicate results from an individual mouse; **a**-**d**) One-way ANOVA. Source data

Flow cytometry conducted to assess ACh generation in the respiratory tract demonstrated that, by frequency and total cell count, most ChAT^+^ leukocytes were among distinct clusters of CD19^+^ B cells (Fig. 3a–d and Extended Data Fig. 2a–g). ChAT^+^ B cells were predominantly CD5^+/−^, CD19^+^, CD43^+^, IgM^hi^ and IgD^lo^ and CD138^−^, thus mostly, albeit not exclusively, B-1 cells (Fig. 3e and Extended Data Fig. 2h–l), consistent with a previous study^27^. Although B-1 cells dominated ChAT expression in the pleural cavity by frequency and total numbers, as a result of their larger total cell numbers, conventional mature B cells outnumbered B-1 cells in the spleen, lung and mediastinal lymph nodes (MedLNs) (Extended Data Fig. 2h–l).

**Extended Data Fig. 3:**
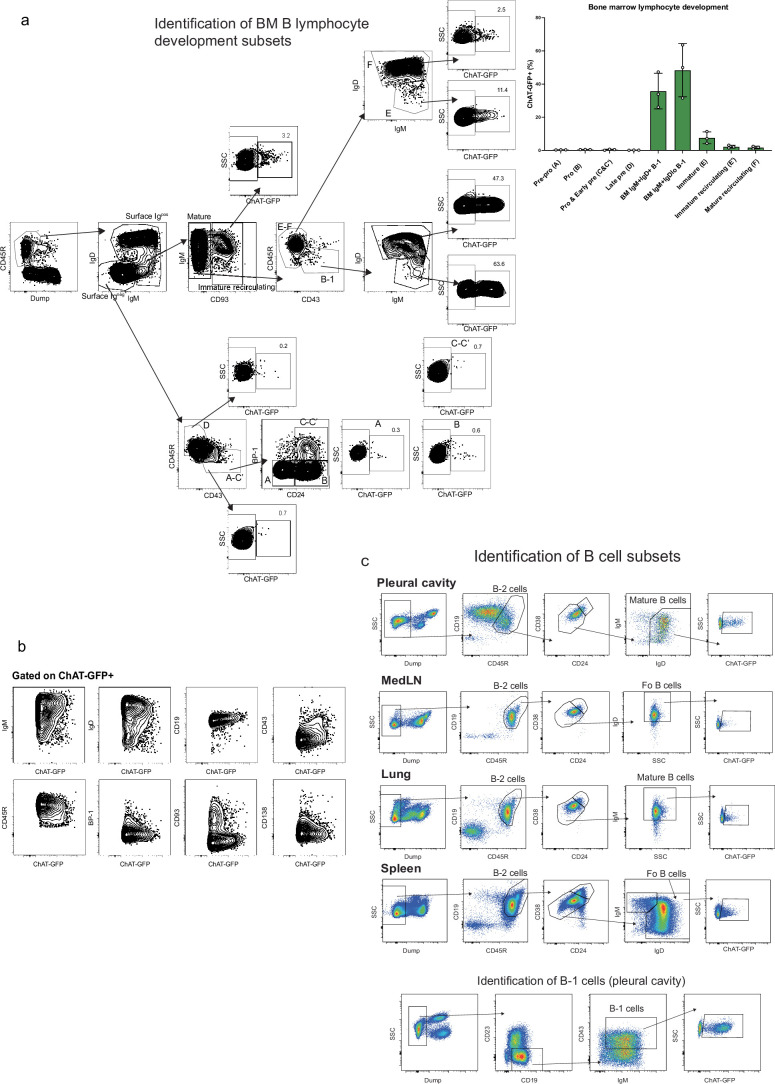
ChAT induction first occurs at the immature B cell stage. Bone Marrow (BM) from ChAT-GFP reporter mice (n = 3) analyzed by flow cytometry to identify ChAT-expression among B cell differentiation stages. **a**) (left) gating strategy using CD45R to identify B cells and separation of cells into surface Ig+ and Ig negative cells (lower panels), which were then divided into CD43+ (Hardy Fr. A-C’) and CD43- (Hardy Fr. D). Surface Ig+ cells were divided into CD93 (immature, Hardy Fr. E) and CD93- cells. The latter were further separated into CD43- mature circulating B cells (Hardy Fr. F), and CD43+ IgMhi IgDlo B-1 cells; (right) quantification of BM ChAT-GFP + B cells as frequency of the indicated B cell subset. **b**) representative flow plots showing the relationship between ChAT-GFP expression and other surface markers used to define B cell developmental stages. **c**) gating strategy to identify different B cell subsets in all tissues analyzed (lungs, pleural cavity, mediastinal lymph nodes and spleen). Results in a) are representative of 2 independent experiments with n = 3 mice each. Bar graphs show mean ± s.e.m. Symbols indicate results from an individual mouse. Source data

In bone marrow (BM), ChAT expression was observed only rarely among pro- and pre-B cells (Hardy fractions A–D) with increases at the immature B cell stage (B220^hi^IgM^+^IgD^−/lo^CD93^+^; Hardy fraction E) (Extended Data Fig. 3a,b). Similar to peripheral tissues, BM cells with a mature, B-1-like cell phenotype (CD45R^+/lo^CD93^−^, CD43^+/−^, IgD^lo^ and IgM^+^) contained the highest frequency ChAT expressers (Extended Data Fig. 3a,b).

**Extended Data Fig. 4:**
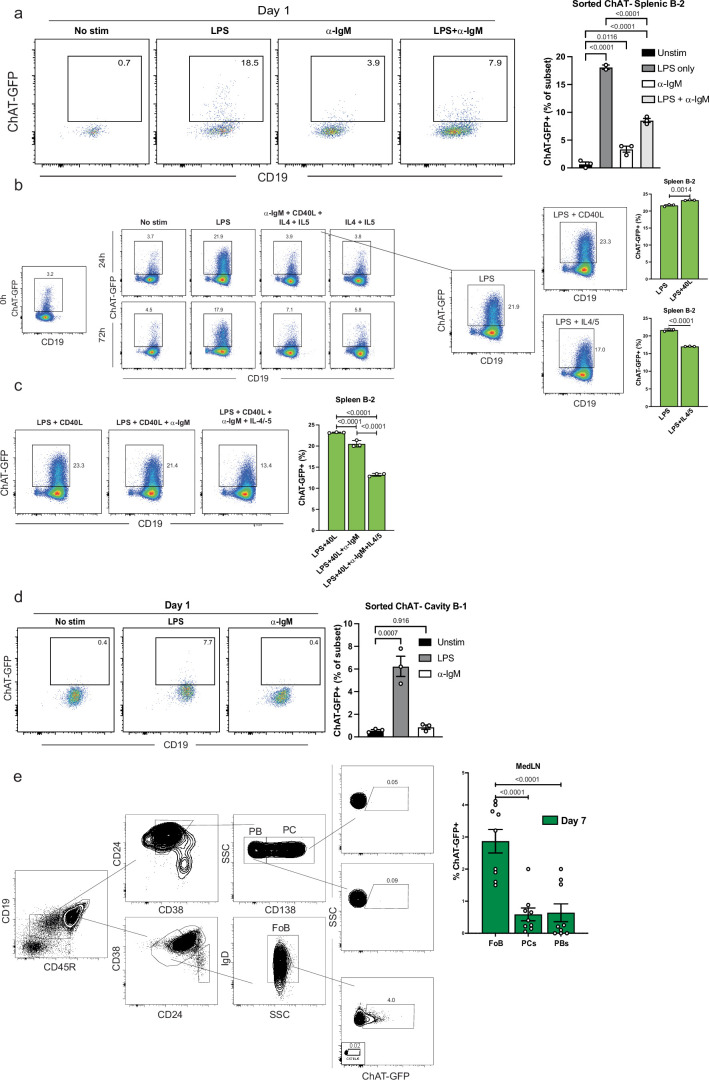
ChAT induction in B cells. **a–d**, flow cytometry plots (left) and frequencies (right) of ChAT-GFP expression among FACS-sorted splenic ChAT-GFP^neg^ B-2 cells (CD19 + ,CD23 + ,CD43-, CD5-, CD9-, ChAT-GFP^neg^) (**a**) or magnetically-enriched total splenic B-2 cells (CD19 + ,CD23 + ,CD43-, CD5-, CD9-) from ChAT-GFP reporter mice cultured in the presence or absence of LPS, anti-IgM (Fab)_2,_ or indicated stimuli, for indicated times (**b**,**c**). **d**, representative flow cytometry plots (left) and frequencies (right) of ChAT-GFP expressing FACS-sorted pleural cavity ChAT-GFP^neg^ B-1 cells (CD19 + , CD23-, CD43 + , ChAT-GFP^neg^) cultured in the presence or absence of LPS, anti-IgM (Fab)_2_, or both, for 24 h. **e**, representative flow cytometry plots and gating strategy (left) and frequencies (right) of ChAT-GFP expressing Follicular B cells (FoB), Plasmablasts (PB) or plasma cells (PC) from MedLN of ChAT-GFP reporter mice infected with 10PFU A/PR8 for 7 days. **a–d**, representative of 2 experiments with similar results; data represent n = 3 technical replicates per group e) n = 9 mice, pooled from 3 independent experiments. Bar graphs indicate mean ± s.e.m. **a–e**, One-Way ANOVA. Source data

LPS, but much less so anti-immunoglobulin M (IgM), induced ChAT-GFP in spleen FO B cells, consistent with previous reports^27^ (Fig. 3f and Extended Data Fig. 4a). This may explain the relatively large frequencies of ChAT-GFP^+^ B-1 cells, because they respond more strongly to TLR-mediated signaling compared with conventional B cells and require MyD88 expression for survival and differentiation^58^. B cell differentiation after stimulation with LPS plus IL-4 and IL-5, as well as stimulation with anti-IgM, CD40L, IL-4 and IL-5 reduced ChAT expression (Extended Data Fig. 4b,c). Similar results were obtained with sorted ChAT^−^ B-1 cells (Extended Data Fig. 4d). Consistent with these findings, plasmablasts or cells obtained from respiratory tract draining MedLNs of ChAT-GFP mice at 7 d.p.i. with influenza A/PR8 lacked ChAT-GFP expression (Extended Data Fig. 4e). The data suggest an innate-like, immediate, early role for ChAT-expressing B cells, independent of further differentiation.

## ChAT B cells control lung viral loads after influenza

**Extended Data Fig. 5:**
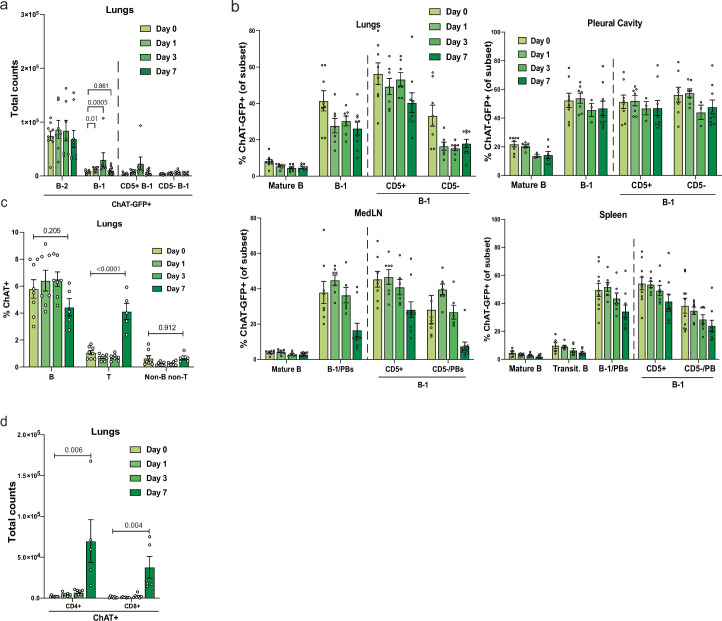
ChAT B cell induction and redistribution after influenza infection. **a**-**c**) Flow cytometry of lungs, pleural cavity, MedLN and spleen of ChAT-GFP reporter mice infected with 10PFU A/PR8 for 0, 1, 3 and 7 days. **a**) total cell counts of ChAT-GFP + B cell subsets (Extended Data Fig. 2c) (right). **b**) frequency of ChAT-GFP+ expressing cells among each B cell subset in lungs, pleural cavity, spleen and MedLN **c**) frequency of ChAT-GFP + B (CD19 + ), T-ILC (CD3+ or Thy1.2 + ) and Non-B/Non-T (CD19-, CD3-, Thy1.2-) among total ChAT-GFP+ cells. **d**) cell counts of ChAT-GFP + CD4+ and CD8 + T cells in the lungs at indicated days after influenza infection. **a**-**d**) n = 8/lung and Pleural cavity; n = 8/MedLN and n = 10/spleen mice pooled from 3 independent experiments. Bar graphs show mean ± s.e.m. Symbols indicate results from an individual mouse. **a**-**d**) One-Way ANOVA. Source data

A role for B cell-derived ACh was revealed after influenza infection when viral loads were assessed in mice with a B cell-specific deletion of ChAT (mb-1Cre^+/−^ChAT^fl/fl^ mice, ChatBKO). At 1 d.p.i. ChatBKO mice had tenfold reduced lung viral loads compared with mb-1Cre^−/−^ChAT^fl/fl^ controls (Control, Fig. 3h). Although leukocyte-derived ACh effects in the spleen have been shown to require T cell-mediated ACh^51,52^, T cell-specific deletion of ChAT (Chat^fl/fl^-Lck^Cre+/−^) did not affect influenza virus loads at this time (Fig. 3i), consistent with their low frequencies before infection (Extended Data Fig. 2) and also consistent with B cells remaining the predominant population of ChAT-GFP^+^ cells at early infection time points (Extended Data Fig. 5a–e). In addition, significant increases in absolute, but not relative, numbers of ChAT-GFP^+^ B cells were found at 1 and 3 d.p.i. in the lungs of ChAT-reporter mice (Fig. 3g and Extended Data Fig. 5a–c). ChAT-GFP^+^ T cells did not accumulate in the lungs until 7 d.p.i. (Extended Data Fig. 5d), a time when lung virus loads are already greatly reduced. Thus, innate signal-induced ChAT^+^ B but not ChAT^+^ T cells rapidly respond to affect influenza A virus replication in the lung within the first 24 h of infection.

**Fig. 4:**
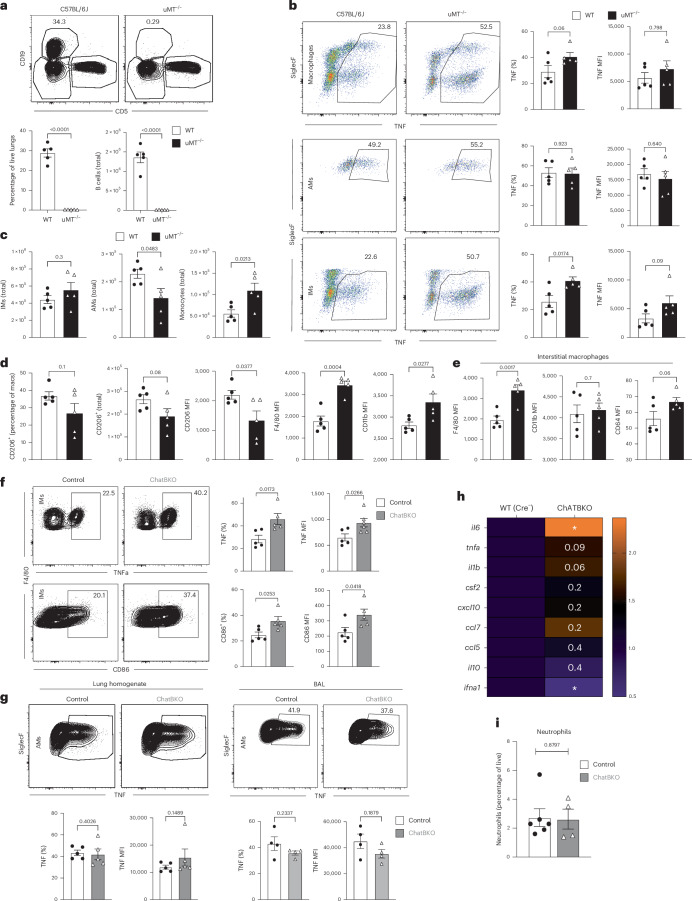
B cells control lung IM responses via ACh. **a**, Representative flow cytometry plots (top) and frequencies (bottom) of CD19^+^, CD5^−^ B cells in the lungs of WT C57BL/6 and μMT^−/−^ mice. **b**–**e**, Flow cytometry on lungs of WT C57BL/6 and μMT^−/−^ mice (*n* = 5) infected with A/PR8 at 1 d.p.i. **b**, Representative flow cytometry plots (left) and frequencies and MFI (right and far right, respectively) of TNF-expressing lung total macrophages (top: CD19^−^, ThY1.2^−^, Ly6G^−^, Ly6C^−^ and F4/80^+^/CD64^+^), AMs (middle: further gated on CD11b^−^, CD11c^+^ and SiglecF^+^) and IMs (bottom: further gated on CD11b^+^, CD11c^−^ and SiglecF) after ex vivo restimulation with LPS. **c**, Total counts of lung IMs (left), AMs (middle) or monocytes (right) gated as in **b**. **d**,**e**, Frequencies and total number of lung parenchyma CD206^+^ macrophages and MFI of indicated markers among total macrophages (**d**) and IMs (**e**). **f**, Representative flow plots (left) and frequencies (right) of TNF-expressing IMs from lung parenchyma gated as in **b** from mb-1Cre^−/−^ChAT^fl/fl^ (control) and mb-1Cre^+/−^ChAT^fl/fl^ (ChatBKO) mice at 1 d.p.i. after infection with A/PR8, following ex vivo restimulation with LPS. **g**, Representative flow plots of TNF production by lung tissue homogenates (left) and BAL (right) AMs from mice as in **g** after ex vivo LPS stimulation. **h**, Relative gene expression of indicated cytokines and chemokines in lung homogenates from control and ChatBKO mice at 1 d.p.i. **i**, Frequency of lung neutrophils (CD19^−^, Thy1.2^−^, Ly6C^−^ and Ly6G^+^) at 1 d.p.i. in control (*n* = 6) and ChatBKO (*n* = 4) mice. In **a**–**h**, *n* = 5 were mice pooled from two independent experiments. The bar graphs show the mean ± s.e.m. The symbols indicate the results from an individual mouse. In **a**–**h**, the two-tailed, unpaired Student’s *t*-test was used. Source data

Given the decreased early control of influenza virus replication in mice in which TNF signaling was blocked (Fig. 2b), the effects of B cells on control of macrophage function were evaluated first by measuring TNF production in B cell-deficient μMT^−/−^ mice (Fig. 4a). IMs but not AMs of lung homogenates of control and μMT^−/−^ mice at 1 d.p.i. with A/PR8 showed significant increases in TNF production (Fig. 4b). The μMT^−/−^ mice also showed increased lung monocyte infiltration and decreased AM numbers, suggesting that the absence of B cells caused enhanced inflammation and perhaps increased AM apoptosis (Fig. 4c)^59–61^. Furthermore, μMT^−/−^ mice had reduced frequencies of inhibitory receptor CD206-expressing macrophages and decreased CD206 expression levels, but higher surface expression of activation markers F4/80, CD11b and CD64 (Fig. 4d) and in IMs (Fig. 4e). Thus, B cells regulate the activation state of IMs, but not AMs, in the respiratory tract immediately early after influenza virus infection.

To determine the extent to which these were the effects of ACh secretion by B cells, A/PR8-infected ChatBKO mice were analyzed at 1 d.p.i. ChatBKO-derived IMs showed increased TNF production as well as increased expression of CD86, whereas AMs isolated from lung homogenates or bronchoalveolar lavage (BAL) were unaffected (Fig. 4f,g). Moreover, lung homogenates from ChatBKO showed increased expression of proinflammatory cytokine genes *il6*, *tnfa*, *il1b* and *csf2*, whereas *il10* showed minimal changes compared with controls. The exression of *ifna1* was reduced in the absence of B cell-derived ACh, consistent with reductions in lung viral loads (Fig. 4h). Depletion of ChAT in B cells, however, did not significantly affect neutrophil numbers at 1 d.p.i., cells that were affected by B cell-derived ACh in a sepsis model (Fig. 4i)^27^. Thus, ChAT B cells modulate the activation and inflammatory cytokine elaboration of lung IMs but not AMs early during influenza infection.

## ChAT B cell activity modulates later inflammatory responses

**Fig. 5:**
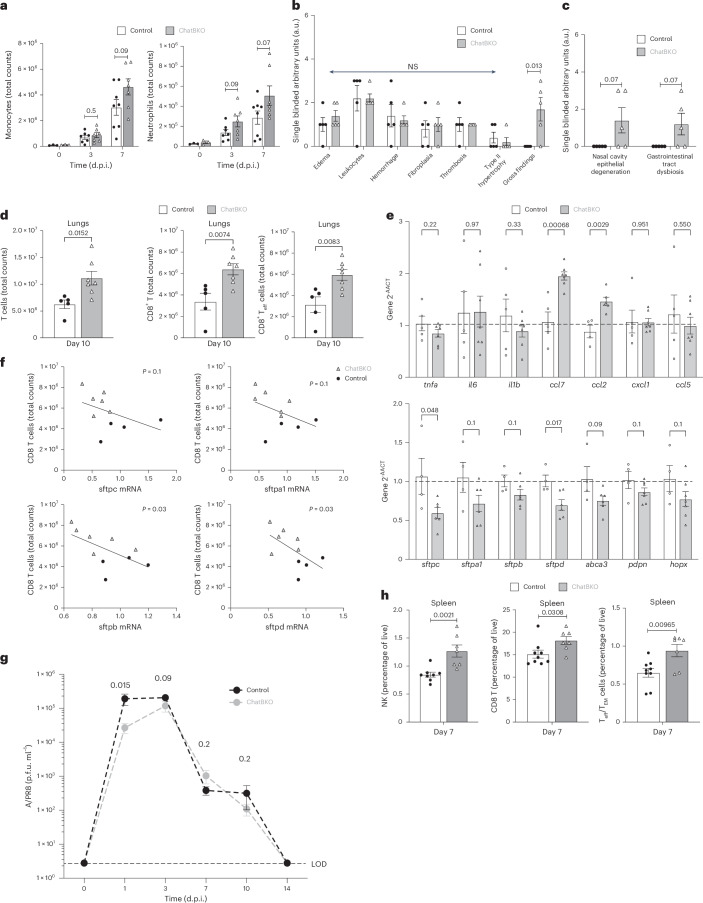
Lack of B cell-derived ACh causes increased local and systemic inflammation. **a**, Total cell counts of monocytes (CD19^−^, Thy1.2^−^, Ly6G^−^ and Ly6C^+^) and neutrophils (CD19^−^, Thy1.2^−^, Ly6C^−^ and Ly6G^+^) of lung homogenates from mb-1Cre^−/−^ChAT^fl/fl^ (control) (*n* = 8) and mb-1Cre^+/−^ChAT^fl/fl^ (ChatBKO) mice (*n* = 7) infected for indicated times with influenza A/PR8. **b**,**c**, Histopathological evaluation of lung parenchyma (**b**) and nasal cavity and gastrointestinal tract (**c**) from indicated mice at 7 d.p.i. after infection with 100 p.f.u. of A/PR8. **d**, Total numbers of T cells (right), CD8 T cells (CD3^+^, CD4^−^ and CD8^+^) (middle) or effector (T_eff_)/effector memory (T_EM_) (CD3^+^, CD4, CD8^+^, CD44^hi^ and CD62L^−^) CD8 T cells in the lung homogenates from control (*n* = 5) and ChatBKO (*n* = 7) mice at 10 d.p.i. infected as in **a**. **e**, Fold-change gene expression in lung homogenates from control (*n* = 5) and ChatBKO (*n* = 7) mice at 10 d.p.i. with A/PR8 compared with noninfected. **f**, Correlations of CD8 T cell numbers and gene expression in lung homogenates. **g**, Lung virus loads at indicated timepoints in control and ChatBKO mice (*n* = 6–12 per group). **h**, Frequencies of spleen NK cells (left; CD19^−^, Th1.2^−^, Ly6G^−^, Ly6C^−^ and NK1.1^+^), CD8^+^ T cells (middle) and effector/effector memory CD8^+^ T cells (right) in control (*n* = 8) and ChatBKO mice (*n* = 7) at 7 d.p.i. with 100 p.f.u. of A/PR8. In **a** and **d**–**h**, mice are pooled from two to three independent experiments and, in **b** and **c**, *n* = 5 mice. The bar graphs show the mean ± s.e.m. The symbols indicate results from an individual mouse. In **a**–**e**, **g** and **h**, two-tailed, unpaired Student’s *t*-tests were used. a.u., arbitrary units. Source data

Early control of virus replication and IM activation by ChAT B cells affected the course of influenza infection. By 7 d.p.i., increases in lung monocyte and neutrophil numbers were consistently noted in ChatBKO mice, although those increases did not reach statistical significance (Fig. 5a). Histopathological evaluation suggested enhanced epithelial degeneration in the nasal cavities and poorer overall health of ChatBKO mice compared with the controls at 7 d.p.i. (Fig. 5b,c). Furthermore, ChatBKO mice showed significant increased lung CD8 T cell infiltration at 10 d.p.i. This correlated with increased *ccl2* and *ccl7* lung mRNA transcript levels and decreased expression of genes associated with epithelial cell wall function and alveolar epithelium integrity^62–65^ (Fig. 5d,e). Surfactant protein genes were reduced by up to 50% in the absence of B cell-derived ACh, suggesting increased epithelial damage and decreased integrity, function and maturation of the alveolar epithelium (Fig. 5e). Similar trends, albeit not reaching significance, were also observed for pdpn, hopx and abca3 (Fig. 5e). Changes indicative of epithelial dysfunction negatively correlated with total lung CD8^+^ T cell counts, highlighting the association between immunopathology and CD8^+^ T cell infiltration (Fig. 5f). Changes at 10 d.p.i. were notable, because. by 7 d.p.i., viral loads were comparable and both groups of mice cleared the infection by 14 d.p.i., despite early viral load differences (Fig. 5g). Increased systemic inflammation with enhanced CD8 T cell activation and NK cell recruitment to the spleen was also noted in ChatBKO mice compared with controls at 7 d.p.i. when using a 10-fold higher dose of infection (Fig. 5h) and suggesting insufficient control of lung inflammation as a main cause of increased pathology and immune cell activation. Thus, early suppression of IM activity by ChAT B cells, while enabling increased early viral replication, helped suppress later systemic and local innate and adaptive lung inflammatory responses and support of lung epithelial repair.

## ChAT B cells regulate lung myeloid cells in the steady state

**Extended Data Fig. 6:**
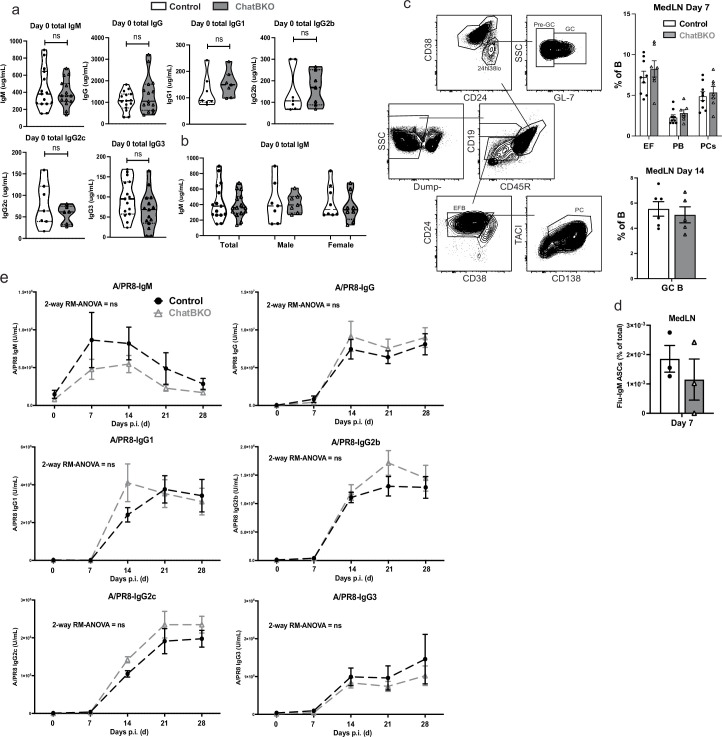
B cell-derived ACh does not alter antibody production. **a**) Serum IgM, IgG, IgG1, IgG2b and IgG2c concentrations in non-infected mb-1Cre^-/-^ ChAT^fl/fl^ (Control) and mb-1Cre^+/-^ ChAT^fl/fl^ (ChatBKO) mice (n = 8-14/group) and **b**) concentrations serum IgM in male and female Control and ChatBKO mice. **c)** Representative flow cytometry plots of MedLN from Control (n = 9) and ChatBKO (n = 7) mice infected with 10PFU A/PR8 for 7 days (left), frequencies of extrafollicular B cells (EF), plasmablasts (PB) or plasma cells (PC) (top-right) and frequencies of germinal center B cells (GC B) at 14dpi (bottom right). **d**) A/PR8-specific IgM ASCs in the MedLN at 7dpi. **e**) Influenza specific serum IgM, IgG, IgG1, IgG2b, IgG2c, IgG3 in Control and ChatBKO mice (n = 9/group). **a**, **b**) Mice pooled from 3 independent experiments **c**) mice pooled from 3 independent experiments. **d**) representative of 2 independent experiments with n = 3 which gave similar results. **e**) results from mice pooled from 2 independent experiments. Bar graphs and violin plots show mean ± s.e.m. Symbols indicate results from an individual mouse. **a**-**d**) two-tailed unpaired Student’s t-test. **e**) repeated measure 2-way ANOVA. Source data

**Extended Data Fig. 7:**
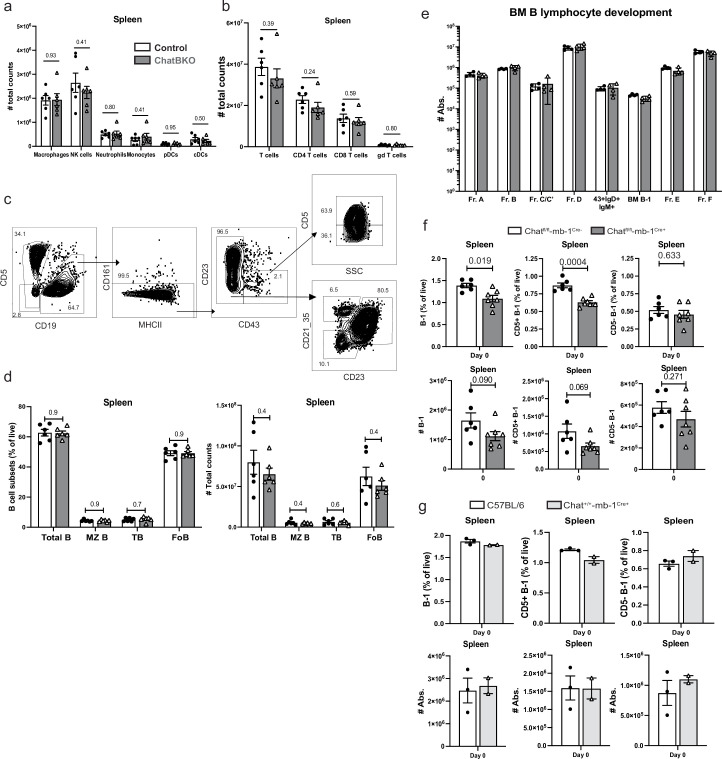
Lack of ChAT B cells does not affect steady state leukocyte composition nor B cell development. **a-e**) flow cytometric analysis of adult male and female mb-1Cre^-/-^ ChAT^fl/fl^ (Control) and mb-1Cre^+/-^ ChAT^fl/fl^ (ChatBKO) mice. **a, b**) cell counts of (**a**) innate leukocytes, and (**b**) T cells. **c**) flow cytometric gating strategy to identify B cell subsets in the spleen. **d**) frequency and absolute numbers of B cell subsets in spleen steady state comparing Control and ChatBKO mice. **e**) Bone Marrow (BM) B lymphocyte development subsets using the Hardy gating scheme as in (Extended Data Fig. 2). **f**) frequencies (top) and total numbers (bottom) of B-1 cells and CD5+ and CD5- B-1 cells comparing Control and ChatBKO mice. **g**) similar to (**f**) but comparing C57BL/6 and mb-1Cre^+/-^ ChAT^+/+^ mice. **a**-**d**, **f**) n = 6 mice pooled from 2 independent experiments. **e**, **g**) representative of 2 independent experiments with similar results, n = 4 each. Bar graphs show mean ± s.e.m. Symbols indicate results from an individual mouse. **a**-**g**) two-tailed unpaired Student’s t-test. Source data

Immediate early influenza virus infection is controlled in part by natural IgM, secreted mostly by B-1 cells^66,67^, which we show here to be a ready source of ACh. However, lack of ChAT expression by B cells did not significantly affect total or virus-binding IgM levels (Extended Data Fig. 6a,b). Similarly, the lack of B cell-expressed ChAT had no effect on extrafollicular plasmablast development or the frequencies of germinal center B cells (Extended Data Fig. 6c), influenza-specific IgM or IgG antibody-secreting cells in the MedLNs at 7 and 14 d.p.i. (Extended Data Fig. 6d) or on serum influenza-specific IgM or IgG subclasses over a 4-week time course (Extended Data Fig. 6e). Deletion of ChAT in B cells also did not affect total numbers of innate leukocytes, T or B cell subsets in the spleen or BM B cell development when compared with control mice^68^ (Extended Data Fig. 7a–e). The exception was a slight but significant reduction of CD5^+^ B-1 cells in ChatBKO compared with ChAT^fl/fl^-Cre^−^ mice (Extended Data Fig. 7f). However, nonfloxed, Cre-expressing mb-1^Cre−/−^ChAT^+/+^ mice showed similar reductions, suggesting that this effect was driven by mb-1 haploinsufficiency not ChAT expression (Extended Data Fig. 7g)^69–72^.

**Fig. 6:**
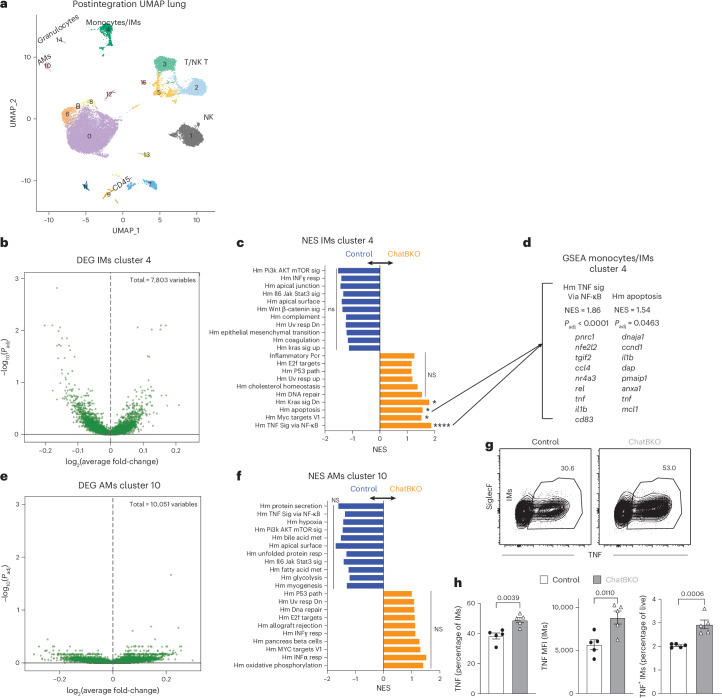
ChAT B cells alter the transcriptional profile of myeloid cells. **a**, ScRNA-seq post-sample integration UMAP plots of lung parenchyma cells pooled from two mb-1Cre^−/−^ChAT^fl/fl^ (control) or two mb-1Cre^+/−^ChAT^fl/fl^ (ChatBKO) female mice analyzed by scRNA-seq. **b**,**e**, Differentially expressed genes (DEGs) among cells in clusters 4 (IMs) (**b**) and 10 (AMs) (**e**), comparing control and ChatBKO mice. **c**,**f**, The 10–11 selected, most differentially expressed, Hallmark (Hm) signaling (sig) pathways in clusters 4 (**c**) and 10 (**f**), comparing normalized enrichment scores (NES) for control (blue, left) and ChatBKO (orange, right) mice as identified by gene set enrichment analysis (GSEA); uv, ultraviolet; dn, down; resp, response; pcr, pathology complete response; path, pathway; met, metabolism. **d,** Calculation of *P*_adj_ values and the determination of the NES for the two pathways exhibiting the most DEGs. Eight or nine genes with the most DEGs between groups within each specified pathway are shown. **g**, Representative flow cytometry plots (top) and quantification (bottom) of TNF-expressing lung IMs (CD19^−^, CD5^−^, CD11b^+^, F4/80^+^/CD64^+^CD11c-, Ly6G^−^, Ly6C^−^ and SiglecF^−^) plotted as a frequency of the previous gate (bottom, left), MFI quantification (bottom, middle) and a frequency of live cells (bottom, right) after short-term restimulation with LPS comparing control and ChatBKO mice (*n* = 5 per group). In **c**, **d** and **f**, fgsea v.1.24.0 was used to run GSEA with gene sets obtained from the Molecular Signatures Database. Features were ranked by −log(*P*) × sign(fold-change). In **g**, pooled mice are from two independent experiments. The bar graphs show the mean ± s.e.m. In **g**, the two-tailed, unpaired Student’s *t*-test was used. Source data

**Extended Data Fig. 8:**
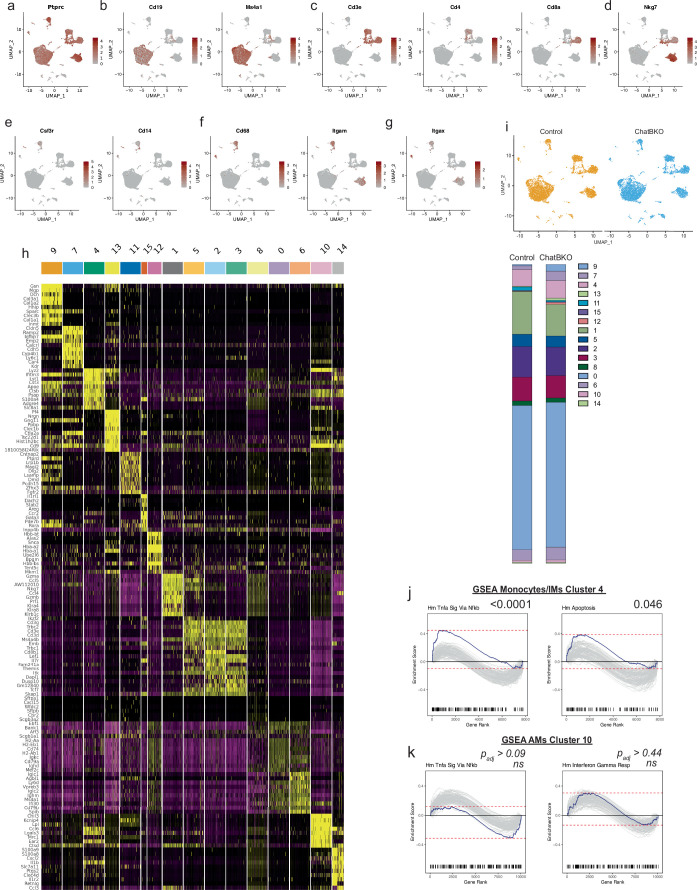
Lack of ChAT B cells alters lung myeloid cells in steady state. **a-g**) scRNA-Seq post-sample integration UMAP analysis plots from live, single cells of lungs from mb-1Cre^-/-^ ChAT^fl/fl^ (Control) and mb-1Cre^+/-^ ChAT^fl/fl^ (ChatBKO) mice (n = 4 females/group). Each graph indicates expression levels of indicated canonical cell lineage markers. The intensity of expression is indicated by red coloring. **h**) Heat map of post-sample integration gene clustering analysis indicating the ten most differentially expressed genes across all clusters. **i**) (top) scRNA-Sequencing pre-sample integration UMAP plots of lung parenchyma cells from Control (orange) and ChATBKO (blue) mice, and (bottom) frequencies of cells by cluster and sample, compared to the total number of cells per sample plotted as a stacked bar chart. **j**) scRNA-Seq hallmark Tnfa Signaling via Nfkb and apoptosis pathway analyses for cluster 4 (IMs) and **k**) hallmark Tnfa Signaling via Nfkb and interferon gamma response pathways for cluster 10 (AMs). **j**-**k**) two-tailed student’s t-test. Source data

To broadly assess the impact of B cell-specific ACh, we conducted single-cell RNA sequencing (scRNA-seq), comparing cells pooled from two lungs per group of control and ChatBKO mice before infection. Post-sample integration analysis revealed 16 distinct cell clusters (Fig. 6a). Clusters 7, 9, 11 and 13 were CD45^−^ nonimmune cells and the remainder were CD45^+^ leukocytes (Extended Data Fig. 8a and Supplementary Data 1). B cells were identified in clusters 0, 6, 8 and 12, whereas T or NK T cells were present in clusters 15, 5, 2 and 3 and NK cells in cluster 1 (Extended Data Fig. 8b–d and Supplementary Data 1). Clusters 10, 14 and 4 represented myeloid cells (Extended Data Fig. 8e–h and Supplementary Data 1). Cluster 10 exhibited markers indicative of AMs, cluster 14 granulocytes and cluster 4 are monocytes and monocyte-derived interstitial macrophages (IMs) (Extended Data Fig. 8e–h) expressing markers of activation, including *tnf*, *socs3*, *cd80* and *cd86* (Supplementary Data 1). Lung cell subset frequencies were similar between control and ChatBKO mice (Extended Data Fig. 8i).

Among CD45^neg^ clusters, only cluster 11 showed significant increases in gene pathways associated with interferon (IFN)γ responses, IFNα responses and the PI3K/AKT^−^/mTOR (mammalian target of rapamycin) signaling pathway in lungs of ChatBKO compared with control mice (Supplementary Data 2). Among the CD45^pos^ clusters, most B cells (cluster 0, 6 and 8) and CD8 T cells (cluster 2) lacked significant differences (Supplementary Data 2), whereas NK and NK T cells (clusters 1 and 3) showed a lower TNF signaling via the NF-κB pathway (Supplementary Data 2). Among the myeloid clusters, AMs (cluster 10) showed no differences, whereas granulocytes (cluster 14) and the biggest myeloid cluster (cluster 4), consisting of monocytes and IMs, showed the strongest differences between control and ChatBKO mice with statistically significant differences in expression levels of 700 genes (Fig. 6b, Extended Data Fig. 8j and Supplementary Data 2). Gene set expression analysis (GSEA) demonstrated upregulation of several hallmark pathways in IMs of ChatBKO mice, including *kras* signaling, *myc* targets V1 and, notably, the apoptosis and the *tnfa* signaling via *nfkb* pathways (*P*_adj_ < 0.05 and *P*_adj_ < 0.0001) (Fig. 6c,d). The apoptosis genes *dap*, *pmaip1*, *anxa1* and *mcl1*, and activation and immune-related genes *pnrc1*, *nfe2l2*, *ccl4*, *rel*, *tnf*, *il1b* and *cd83* were genes driving the difference, consistent with the flow cytometric data, suggesting that inhibition of viral replication via TNF may occur through increased apoptosis^73,74^. Again, the AM cluster cells showed no significant differences between the mouse groups (Fig. 6e,f and Extended Data Fig. 8k). Thus, B cell-derived ACh specifically impacts IM and myeloid cells in the lung.

**Extended Data Fig. 9:**
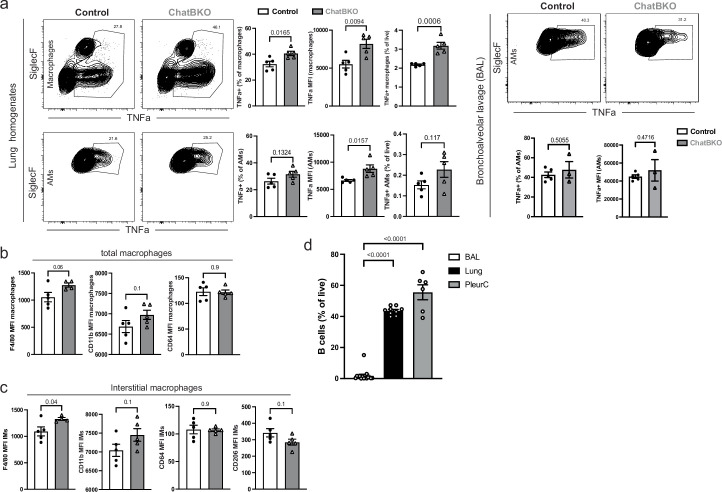
B cell derived ACh inhibits lung interstitial- but not alveolar- macrophage activation. **a-c**) flow cytometry of single cell suspensions from lung parenchyma (left) and BAL (right) of mb-1Cre^-/-^ ChAT^fl/fl^ (Control) and mb-1Cre^+/-^ ChAT^fl/fl^ (ChatBKO) mice. **a**) representative flow cytometry plots (left) and frequencies (right) of TNFα-expressing lung or BAL macrophages (top; CD19-, ThY1.2-, Ly6G-, Ly6C-, F4/80 + /CD64 + ) and alveolar macrophages (bottom; AMs; CD19-, ThY1.2-, Ly6G-, Ly6C-, F4/80 + /CD64 + CD11c + , CD11b-, SiglecF + ), frequencies are of previous gate (% of macrophages), TNFα shown as frequencies of live, MFI and as a frequency of macrophages, respectively, after ex-vivo LPS restimulation in the presence of Brefeldin A for 4 h at 37 C. **b**) MFI of indicated markers (F4/80, CD11b, CD64) in total macrophages and **c**) in IMs. **d**) frequency of B cells in BAL (n = 12), lung parenchyma (n = 9) and pleural cavity (n = 6) cells represented as a frequency of live events from C57BL/6 mice. **a**-**c**) n = 5 mice pooled from 2 independent experiments with 2-3 mice each. **d**) mice pooled from 3 independent experiments. Bar charts show mean ± s.e.m. Symbols indicate results from an individual mouse. Two-tailed unpaired Student’s t-test. Source data

Consistent with these findings, IMs from noninfected ChatBKO mice had reduced TNF production compared with controls after short-term restimulation (Fig. 6g and Extended Data Fig. 8a–c). The scRNA-seq data confirmed that changes among lung macrophages were restricted to IMs but not AMs, with AMs isolated from either lung or BAL showing similar frequencies and total numbers of TNF producers between noninfected ChatBKO and controls (Fig. 6g and Extended Data Fig. 9a–c) and significant increases in F4/80 expression on IMs and subtle differences in surface expression of CD11b and CD206 (Extended Data Fig. 9b,c) in cells from ChatBKO mice. These differential effects of B cells on IMs and AMs are explained by their virtual absence from the airways of mice (Extended Data Fig. 9d)^75,76^.

## ChAT B cells inhibit IMs via α7nAchR

**Fig. 7:**
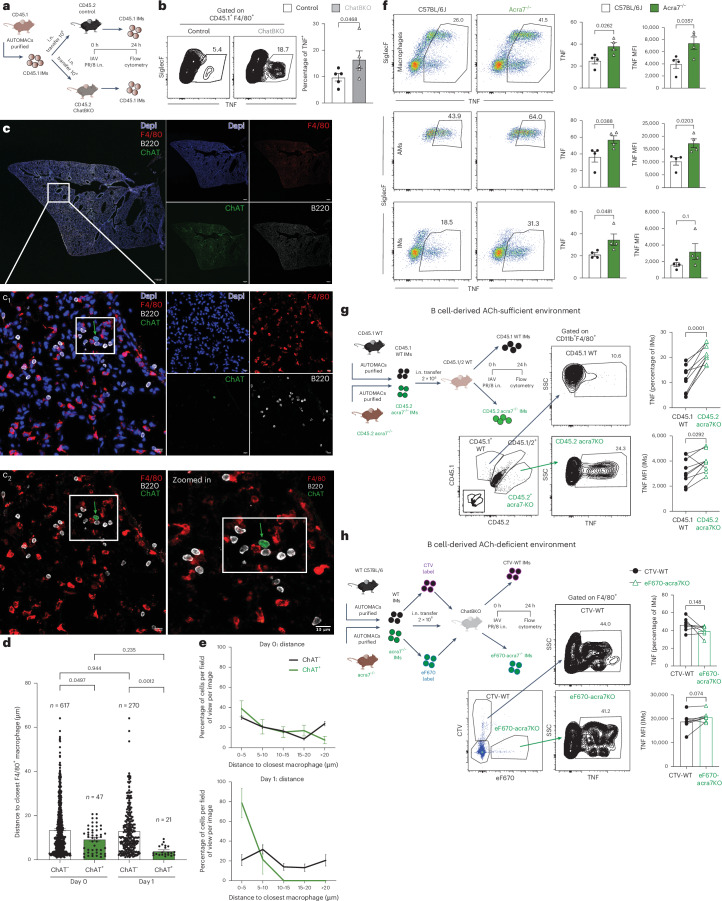
B cells modulate lung IMs via ACh and α7nAChR. **a**, Experimental design. **b**, Representative flow plots (left) and frequencies (right) of CD45.1 TNF-producing lung CD45.1^+^, CD45.2^−^ and F4/80^+^ IMs (*n* = 5 per group). **c**, Maximum projection lung immunofluorescent images (×10) of ChAT-GFP mice at 1 d.p.i. with A/PR8. Scale bar, 500 μm. Right, single channels. Boxes show the area of focus. **c**_1_, The ×63 oil *z*-stack maximum projection image. Scale bar, 10 μm. Right, single channels. Boxes show the area of focus and arrows indicate ChAT^+^ B cells in the vicinity with IMs. **c**_2_, Lung immunofluorescent image from **b** zoomed in (right). Scale bar, 10 μm. **d**, Distance (in μm) of indicated B cells (*n* = 955) to nearest macrophage. **e**, Frequency of indicated B cells per field of view binned by distance to closest macrophage, before (top) and at 1 d.p.i. (bottom) with A/PR8. **f**, Left, representative flow plots. Right, frequencies and MFI (right and far right) of TNF by: top, CD19^−^, Thy1.2^−^, Ly6G^−^, Ly6C^−^ and F4/80^+^/CD64^+^ macrophages; middle, AMs (further gated on CD11b^−^, CD11c^+^ and SiglecF^+^); and bottom, IMs (further gated on CD11b^+^, CD11c^−^ and SiglecF^−^) from lungs of 24-h A/PR8-infected WT C57BL/6 and *acra7*^−/−^ mice (*n* = 4). **g**, Left, experimental design. Right and bottom, representative flow plots from lung macrophages gated on CD45 allotype to identify WT (CD45.1) (*n* = 9), *acra7*^−/−^ (*n* = 8) mice (CD45.2) and host (CD45.1/2) cells stained for TNF. Far right, frequencies of TNF (top) and MFI (bottom); paired cells from the same recipient. **h**, Left, experimental design. Middle, gating strategy identifying differentially dye-labeled WT C57BL/6 (CTV) (*n* = 7) and *acra7*^−/−^ (*n* = 6) lung IMs 24 h after adoptive transfer into influenza A/PR8-infected ChatBKO mice. Right, frequencies of TNF^+^ lung IMs. In **a**, **b** and **f**, two representatives of independent experiments are shown with similar results and, in **c**, representatives of three independent experiments with similar results. In **d** and **e**, the total number of cells analyzed is shown from >30 images across 10 slides from two mice per timepoint and, in **g** and **h**, mice pooled from two independent experiments are shown. The bar graphs show the mean ± s.e.m. In **a** and **b**, the one-tailed, unpaired Student’s *t*-test is used, in **d**, the one-way ANOVA and, in **f**, the two-tailed, unpaired Student’s *t*-test. Source data

Given the extremely short half-life of ACh, and the apparent need for co-localization of B cells with macrophages, the data suggested that ChAT^+^ B cells exert a direct effect on IMs. To assess this further, lung IMs from CD45.1^+^ C57BL/6 wild-type (WT) mice were enriched to >75% by magnetic cell separation and adoptively transferred i.n. into CD45.2^+^ C57BL/6 WT controls and CD45.2^+^ ChatBKO, which were then infected for 24 h with influenza A/PR8 (Fig. 7a). Significantly greater TNF responses after short-term restimulation were seen from the transferred IMs placed into ChatBKO compared with those placed into control mice (Fig. 7b), thus ruling out differences in IM development and/or epigenetic changes in ChatBKO mice as reasons for their enhanced inflammatory responses. Furthermore, confocal microscopy revealed co-localization of B220 (CD45R)^+^ B cells and F4/80^+^ macrophages in the lung interstitium of ChAT-GFP reporter mice at 1 d.p.i. (Fig. 7c–e and Supplementary Data 3). GFP^+^ChAT B cells were often seen among small clusters of GFP^−^ B cells and in close proximity to one or more F4/80^+^ macrophages (Fig. 7c and Supplementary Data 3). ChAT^+^ B cells were closer to F4/80^+^ macrophages on average compared with ChAT^−^ B cells and there was a slight, albeit nonsignificant, decrease in the average distance between B cells and macrophages at 1 d.p.i. (Fig. 7d). More ChAT^+^ than ChAT^−^ B cells were in proximity to macrophages (Fig. 7e). Thus, ChAT^+^ B cells seem to be recruited preferentially to F4/80^+^ macrophages, a response that seems to be enhanced after influenza infection.

The inhibitory effects of T cell-derived ACh on splenic macrophages depend on the α7-nicotinic (n)ACh receptor (R) (α7nAChR)^77–83^. In addition to revealing a role for α7nAChR in controlling inflammatory responses of lung macrophages by ACh, increased TNF generation was observed in lung homogenate AMs and IMs from A/PR8-infected *acra7*^−/−^ mice at 1 d.p.i. compared with controls (Fig. 7f).

Additional IM cell adoptive transfer experiments were conducted to probe further for a direct impact of B cell-derived ACh on IMs. First, equal numbers of enriched IMs from CD45.2^+^ *acra7*^−/−^ mice and CD45.1^+^ WT controls were transferred i.n. into WT CD45.1/2 coexpressing C57BL/6 mice, which were then infected with influenza A/PR8 (Fig. 7g). At 1 d.p.i., lung CD45.2^+^ IMs from *acra7*^−/−^ mice demonstrated significantly enhanced TNF production compared with the co-transferred WT CD45.1^+^ cells (Fig. 7g). In contrast, cell tracker dye-labeled WT (CTV) and *acra7*^−/−^ (eF670) CD45.2^+^ IMs, when co-transferred into ChatBKO mice, which were subsequently infected for 24 h, showed no significant difference in TNF production (Fig. 7h). We conclude that ACh-producing B cells directly modulate TNF production by lung IMs early after influenza infection.

## Discussion

**Extended Data Fig. 10:**
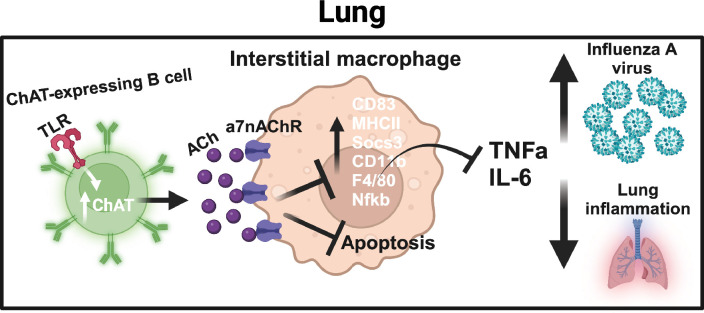
B cell-derived ACh controls lung interstitial macrophage responses to influenza infection. Lung B cells, upon receiving innate-like signals such as through TLR4, induce choline acetyltransferase (ChAT) expression, enabling them to generate acetylcholine (ACh). In both steady-state conditions and immediately following influenza-infection, B cells engage with α7 nicotinic acetylcholine receptor (α7nAChR)+ lung interstitial macrophages (IMs), residing in close proximity. B cell secretion of ACh stimulates IMs via α7nAChR, inhibiting apoptosis and suppressing local inflammation, thereby allowing enhanced early viral replication. This early-stage inhibition of pro-inflammatory cytokine production is beneficial to host organ function and local and systemic inflammation, helping the maintenance and/or repair of lung tissue during and after respiratory tract infection. Figure created using BioRender.com.

Rapid induction of cytokines and chemokines is needed to orchestrate a strong and protective innate immune response. The quality and magnitude of this response not only directly affect pathogen replication, but also significantly affect the long-term consequences of this immune activation, balancing the need for pathogen control with the requirement to avoid host tissue damage. Lung IMs are increasingly recognized as critical components of infection-induced inflammatory cytokine and chemokine responses as well as facilitators of tissue repair. Their controlled activation is thus critical in balancing host immune responses. In the present study, we demonstrated a new regulatory axis by which α7AChR^+^ lung macrophages are regulated by ChAT^+^ lung tissue B cells (Extended Data Fig. 10), the latter co-localizing with macrophages in the lung parenchyma but not the airways. The B cells’ ability to generate ACh seems to directly regulate IM activation and TNF production and, with it, the control of influenza virus replication. Their absence resulted in enhanced elaboration of other proinflammatory cytokines in the lung, increased lung CD8 T cell infiltration, lack of epithelial repair, as well as increased splenic NK cell activation and enhanced clinical pathological signs of disease later in infection.

The two most prominent signaling pathways of lung IMs enhanced in ChatBKO mice were the apoptotic pathway and the TNF signaling via the NF-κB pathway. Treatments targeting TNF and IFNγ have shown promise in reducing mortality by attenuating cytokine storms in both mice and humans^2–4,84–87^, although their precise mechanisms and their long-term effects on lung repair and epithelial integrity remain unclear^5,6,57,88–90^. Furthermore, clinical trials using corticosteroids in patients with COVID-19 highlighted the critical need for carefully balancing inflammation while controlling viral replication^91–98^. Our studies suggest that B cells can provide highly targeted control of lung IM function^9,99–101^.

The study adds to the growing list of immunomodulatory functions attributable to ACh; it also adds another antibody-independent, immune-regulatory function to B cells. ChAT expression by B cells is promoted by TLR4 stimulation, consistent with the dependency of ChAT-expressing B cells on MyD88 (ref. ^27^). However, it is unclear whether TLR stimulation is the only innate signal responsible for ChAT expression by B cells. During activation and plasma cell differentiation, TLR-induced ChAT expression was inhibited, indicating a division of labor, in which antigen-specific B cells remain ChAT^−^ and differentiate instead into antibody-producing plasmablasts and plasmacells, whereas those activated by innate signals, but not stimulation via the B cell receptor (BCR), may be recruited into early innate immune response regulation. This would be consistent with findings that many ChAT^+^ cells belong to the B-1 cell subset, a cell population that does not respond to BCR signaling with clonal expansion^102^, as well as immature B cells, another cell population that does not respond to BCR signaling with activation and differentiation, unless rescued by provision of T cell help or other costimulatory signals^103–105^. Intriguingly, IL-10-producing B cells (B_reg_ cells)^18,106,107^ are also most prevalent among B-1 and immature B cells. However, IL-10 production was also found among certain plasma cells^108–110^.

ChAT^+^ B cells store ACh in cytoplasmic vesicles^68^ from which they can be released rapidly via stimulation with the intestinal neuropeptide cholecystokinin^27^. What regulates ACh release by B cells in the lung parenchyma remains to be defined. It is tempting to speculate that this may involve neuropeptides released by specialized sensory nociceptive neurons and pulmonary neuroendocrine cells in the respiratory tract^25,111^. Of note, we already found differences between the transcriptional profile of various myeloid cell populations in the lungs of ChatBKO mice compared with controls before the influenza virus infection, suggesting the continuous release of ACh from B cells at this site, perhaps triggered by continuous environmental stimuli.

The known cholinergic anti-inflammatory pathway involves ACh receptor (AChR) agonists inhibiting cytokine expression in human macrophages and septic rats and mice, improving survival^77,80–83,112,113^. Later studies emphasized the crucial role of the α7nAChR in inhibiting inflammation and preventing sepsis^78,79,114,115^, effects that have shown efficacy for conditions such as rheumatoid arthritis, inflammatory bowel disease and colitis^35–39^. Given the known role of ACh derived from T lymphocytes in regulating splenic macrophages, the lack of effect of T cell-derived ACh on macrophage function in the lung after influenza infection was somewhat surprising. However, it is consistent with the prevalence of ChAT-expressing leukocytes in the respiratory tract, spleen and MedLNs, both at steady state and at 24 h after influenza infection. Moreover, all ChAT-expressing T cells had an activated CD44^hi^CD62L^lo^ phenotype, a cell population that does not appear in the lungs of infected mice until around 7 d.p.i., revealing profound differences in the kinetics and regulation of ACh production by B and T cells.

In conclusion, our studies indicate that B cell-derived ACh regulates IMs during respiratory tract viral infections, cells that are increasingly identified as critical sources of protective innate, but also harmful inflammatory, responses and misdirected tissue repair. Despite the broad presence of ACh in neuronal synapses in all tissues, and strong expression of the numerous cholinergic receptors on many cell types, its regulatory effect on lung IMs during early infection depends on secretion by B cells that reside in close proximity in the same tissue space, revealing a new, highly cell- and location-specific regulatory axis controlling lung inflammation.

## Methods

### Mice

Male and female 8- to 14-week-old C57BL/6J (CD45.2, cat. no. 000664), C57BL/6J-Ptprc^em6Lutzy^/J (JAXBoy, CD45.1, cat. no. 033076), B6.Cg-Tg(RP23-268L19-EGFP)2Mik/J (ChAT^BAC^eGFP, cat. no. 007902), B cell-deficient (uMT, cat. no. 002288), B6;129-Chat^tm1Jrs^/J (ChAT^flox^, cat. no. 016920), B6-C(Cg)-Cd79a^tm1(cre)Reth^/EhobJ (Mb1-Cre on C57BL/6, cat. no. 020505), B6.Cg-Tg(Lck-cre)548Jxm/J (Lck-Cre 548-O and cat. no. 003802) B6.129S4-*Ccr2*^*tm1Ifc*^/J (ccr2^−/−^, cat.no. 004999) mice were commercially obtained from the Jackson Laboratories.

Strains B6;129-Chat^tm1Jrs^/J (ChAT^flox^, cat. no. 016920), B6-C(Cg)-Cd79a^tm1(cre)Reth^/EhobJ (Mb1-Cre on C57BL/6, cat. no. 020505) and B6.Cg-Tg(Lck-cre)548Jxm/J (Lck-Cre 548-O, cat. no. 003802) mice were initially provided by C. Reardon and K. Murray (University of California, Davis (UC Davis)) and then continued to be bred in the animal facilities at Johns Hopkins University.

B6;129-Chat^tm1Jrs^/J (ChAT^flox^, cat. no. 016920) mice were bred with B6-C(Cg)-Cd79a^tm1(cre)Reth^/EhobJ (Mb1-Cre on C57BL/6, cat. no. 020505) mice to generate a B cell-specific deletion of *chat* and with B6.Cg-Tg(Lck-cre)548Jxm/J (Lck-Cre 548-O, cat. no. 003802) mice to generate a T cell-specific deletion of *chat*. C57BL/6J (CD45.2, cat. no. 000664) and C57BL/6J-Ptprc^em6Lutzy^/J (JAXBoy CD45.1, cat. no. 033076) mice were bred to generate a CD45.1/2 strain for adoptive transfer experiments.

All mice were housed in specific pathogen-free conditions in ventilated filtertop cages with freely available food and water at UC, Davis and Johns Hopkins Bloomberg School of Public Health. Mice were housed on a 6:30–21:00 on:off light cycle, with 20–26 °C and 30–70% humidity standards. Euthanasia was carried out by overexposing mice to CO_2_. All studies involving mice were conducted in compliance with, and after approval of, protocols by the UC Davis Institutional Animal Care and Use Committee (IACUC) and the Johns Hopkins University ACUC.

### Influenza infections

Mice were anesthetized and infected intranasally (i.n.) with influenza A/Puerto Rico/8/34 (A/PR8) virus; 10 plaque-forming units (p.f.u.) per mouse were used, unless otherwise stated in the figure legend, in 40 μl of phosphate-buffered saline (PBS)^116^, which was established as a sublethal dose generating on average <20% body weight loss over the course of infection.

### Tissue processing and flow cytometry staining

Lymph node and spleen cell suspensions were prepared as previously outlined^116^. Briefly, tissues were ground between the frosted parts of two microscope slides and then incubated in ammmonium–chloride–potassium lysing buffer for 1 min on ice to eliminate erythrocytes. Subsequently, the cells were passed through a 50-μm nylon filter and diluted for staining.

For lung tissue collection, lungs were harvested after left ventricle perfusion of the heart and then mechanically and chemically digested. The lungs were placed in 3 ml of Dulbecco’s modified Eagle’s medium (DMEM) F12 1× with 10% NCS in gentleMACS M Tubes (Milentyi, cat. no. 130-093-236) and processed using a gentleMACS dissociator m_Lung_02 twice (Miltenyi). After this, the lungs were incubated with DNAse I (50 U ml^−1^; Worthington-Biochem, cat. no. LS002139) and collagenase, type I (250 U ml^−1^; Worthington-Biochem, cat. no. LS004196) for 25 min at 37 °C and shaking at 220 rpm. After incubation, the lungs underwent another round of processing using the m_lung_02 program. The resulting cells were passed through a 50-μm nylon filter and diluted for staining, similar to lymph node and spleen preparations.

Single-cell suspensions were incubated with Fc receptor block (anti-CD16/32, made in-house) and Live/Dead Fixable Aqua or Near IR (Thermo Fisher Scientific, cat. no. L34967 or L34994) in PBS for 20 min on ice. Subsequently, the cells were stained with fluorescently labeled, anti-surface receptor antibodies according to the manufacturer’s instructions with regard to temperature and duration (see Supplementary Data for the list of antibodies used) in staining medium^116^. All fluorophore-conjugated antibodies were titrated before use to ensure maximal differential staining between the negative and positive fractions. For intracellular staining, the eBioscience Foxp3 Transcription Factor Staining Buffer Set (Thermo Fisher Scientific, cat. no. 00-5523-00) was used following the manufacturer’s instructions. Data were collected on a BD FACSymphony A3 using FACS Diva v.8.0.3 software.

### Magnetic cell enrichment (auto-MACS) and ex vivo cell cultures

Single-cell suspensions from the lung or spleen were prepared as described above and treated with anti-mouse CD16/32 (10 μg ml^−1^) for 20 min on ice to block Fc receptors. Subsequently, the cells were stained with biotinylated anti-surface receptor antibodies according to the manufacturer’s instructions about temperature and duration, in the staining medium. Anti-biotin microbeads were then added to the cells according to the manufacturer’s protocol and the cells were passed through a 50-μm nylon filter before separation using the auto-MACS Pro Separator with the ‘depletes’ option (Miltenyi Biotec, cat. no. 130-092-545). IMs from the lung were purified using anti-biotin magnetic beads after staining cells with biotinylated anti-CD90.2 (30-H12), -CD4 (GK1.5), -CD5 (53-7.3), -CD8a (53-6.7), -Gr-1 (RB6-8C5), -NK1.1 (PK136) and -CD49b (DX5), resulting in 70–75% purities as determined by subsequent flow cytometry analysis. Splenic B-2 cells were purified using biotinylated anti-CD90.2 (30-H12), -CD4 (GK1.5), -CD8a (53-6.7), -Gr-1 (RB6-8C5), -NK1.1 (PK136), -CD11b (M1/70), -F4/80 (BM8), -CD5 (53-7.3), -CD9 (MZ3) and -CD138 (281-2), and peritoneal cavity B-1 cells were purified using biotinylated anti-CD90.2 (30-H12), -CD4 (GK1.5), -CD8a (53-6.7), -Gr-1 (RB6-8C5), -NK1.1 (PK136), -CD23 (B3B4), -F4/80 (BM8), -CD5 (53-7.3), -CD9 (MZ3) and -CD138 (281-2). The purity of B cells was >95%, as determined by subsequent flow cytometry analysis.

#### B cell cultures

Auto-MACS-enriched, splenic B-2 or pleural/peritoneal cavity B-1 cells were cultured at 5 × 10^6^ cells ml^−1^ per well or 1 × 10^6^ cells ml^−1^ per well, respectively, in 100 μl of culture medium containing Roswell Park Memorial Institute-1640 medium (Gibco, cat. no. 21870076), penicillin–streptomycin–glutamine (Gibco, cat. no. 10378016), FBS (Gibco, cat. no. 16140071) and 2-mercaptoethanol (Gibco, cat. no. 21985-023) in 96-well round-bottomed plates (Falcon, cat. no. 353077) and stimulated for designated times at 37 °C with 5% CO_2_. Cells were analyzed via flow cytometry with fluorescently labeled antibodies after Fc blocking with anti-CD16/32 (in-house) and Live/Dead staining.

#### Macrophage cultures and ex vivo restimulation

Auto-MACS-purified lung IMs or pleural/peritoneal cavity macrophages were cultured at 1 × 10^6^ cells ml^−1^ per well in 100 μl of culture medium as described above in 15-ml conical tubes and stimulated for designated times at 37 °C with 5% CO_2_. Cells were analyzed via flow cytometry with fluorescently labeled antibodies after Fc blocking with anti-CD16/32 (in-house) and Live/Dead staining. For the detection of TNF expression, total lung suspension or purified designated macrophages were stimulated with 100 ng ml^−1^ of LPS (Sigma-Aldrich, cat. no. L6511) in the presence of brefeldin A (Sigma-Aldrich, cat. no. B6542) for 4 h at 37 °C.

### ScRNA-seq

A lung single-cell suspension was prepared as above and submitted for FACS sorting on live cells (FACSAria) using propidium iodide (Miltenyi Biotec, cat. no. 130-093-233) and cells were concentrated at 1,000 cell μl^−1^ before submitting for 10× scRNA-seq. It was ensured that cell viability was >90% before sequencing submission. For single-cell analysis, automatically called cells were further filtered to ensure usage of high-quality droplets with captured cells. RNA barcodes were filtered on total unique molecular identifier count (>500), feature count (>250 features) and percentage of mitochondrial genes (<25%). Signac v.1.9.0 (ref. ^117^) was used to determine nucleosome signaling and transcription start site enrichment scores. Assay for transposase-accessible chromatin (ATAC) barcodes were filtered on the number of fragments mapping to peak regions (>3,000 and <20,000), percentage of fragments mapping to peak regions (> 5%), nucleosome signal scores (<4) and transcription start site scores (>1).

Seurat v.4.3.0 (ref. ^118^) and Signac v.1.9.0 (ref. ^117^), used for handling of normalization, identification of variable genes, scaling, principal component analysis, uniform manifold approximation and projection (UMAP) dimensional reduction and spiking neural network generation, followed by Leiden clustering, were used for RNA-seq and ATAC–seq data, respectively. Batch effect correction was performed using Harmony^119^. Clusters were identified using a combination of marker genes and differential expression comparing each cluster with all other cells in the dataset. Differential expression analyses were performed using the Mann–Whitney *U*-test. The package fgsea v.1.24.0 (ref. ^120^) was used to run gene set enrichment analysis (GSEA) with gene sets obtained from the Molecular Signatures Database^121^. Features were ranked by −log(*P*) × sign (fold-change).

Percentages of cells by cluster and sample are the percentage of cells in a given cluster, compared with the total number of cells per sample. Statistical comparisons shown are from Student’s *t*-tests comparing the percentages.

Intercellular signaling analyses are based on Domino^122^. UCell^123^ was used to generate transcription factor activation scores by cell using transcription factor target gene sets from the Molecular Signatures Database^121^. ComplexHeatmap v.2.14.0 (ref. ^124^) and circlize v.0.4.15 (ref. ^125^) were used for visualizations.

### Adoptive transfer experiments

WT or *acra7*-deficient IMs were purified using auto-MACS as described above (with purity >75%) and labeled or not with either eF670 (Thermo Fisher Scientific, cat. no. 65-0840-85) or CellTrace Violet (CTV; Thermo Fisher Scientific, cat. no. C34571) following the manufacturer’s instructions. After labeling, the cells were washed with PBS, pooled together and then adoptively transferred into mice i.n. in 20 μl of PBS at the indicated concentrations.

### In vivo treatments

For ACh treatment experiments, acetylcholine chloride (Milipore Sigma, cat. no. A2661) was diluted in 1× PBS (in-house) to specified concentrations and mixed 1:1 with 1 mM of the acetylcholinesterase inhibitor pyridostigmine bromide (PB; Milipore Sigma, cat. no. P9797-1G). An ACh + PB cocktail was then administered in vivo i.n. in 10 μl; PBS was used for the control-treated group.

For TNF blockade experiments, 100 mg of anti-TNF monoclonal blocking antibody^126–128^ (clone XT3.11; Bio X Cell, cat. no. BE0058) was diluted in 1× PBS and administered i.n. in 10 μl or intraperitoneally in 50 μl. Irrelevant rat IgG (clone HRPN; Bio X Cell, cat. no. BE0088) was used as a control-treated group.

### Quantitative real-time PCR and viral RT-qPCR

For cytokine and chemokine mRNA measurement experiments, four small pieces of lung tissue were cut from each lobe before RNA was extracted as per manufacturer instruction (QIAGEN, cat. no. 69504). All samples were compared with a glyceraldehyde 3-phosphate dehydrogenase internal control.

For viral quantitative real-time PCR (RT-qPCR), total lungs were collected and homogenized using gentleMACS M Tubes (Miltenyi Biotec, cat. no. 130-093-236) in 1 ml of PBS, centrifuged and lung homogenate supernatants were collected, aliquoted and snap-frozen. Viral RNA was purified following the manufacturer’s protocol (QIAGEN, cat. no. 52904). All primers were purchased from Thermo Fisher Scientific. The presence of influenza viral RNA was detected via quantification of the influenza AM gene using primers: AM-151 (5′-CATGCAATGGCTAAAGACAAGACC-3′) and AM-397 (5′-AAGTGCACCAGCAGAATAACTGAG-3′) and primer/probe AM-245 (6FAM-5′-CTGCAGCGTAGAGCTTTGTCCAAAATG-3′-TAMRA)^129^. Data were collected in Applied Biosystems QuantStudio 5/6 Flex and quantified using QuantStudio Realtime PCR. Commercial primers for cytokine and chemokine analysis are listed under Supplementary Data.

### ELISA assay

Sandwich ELISA assays were conducted following previously established protocols^130^. In brief, ELISA plates were coated with unlabeled anti-isotype antibodies or whole killed influenza A/PR8/34 (200-400 hemagglutinating units HAU ml^−1^; prepared in-house). To minimize nonspecific binding, the plates were blocked with a blocking buffer containing 1% heat-inactivated newborn calf serum, 0.1% dried milk powder and 0.05% Tween 20 in PBS. Serum obtained from tail bleeds or standards were pre-diluted in PBS and then added to the plate in serial dilutions. Antibody detection was achieved using biotinylated anti-isotype antibodies, followed by streptavidin–horseradish peroxidase (HRP). The reaction was visualized by adding 3,3′,5,5′-tetramethylbenzidine substrate diluted in 0.05 M citric acid and H_2_O_2_ for 10–15 min before stopping with 1 N sulfuric acid. Absorbance was measured at 450 nm and 595 nm on a Molecular Devices SpectraMax M5 and quantified using Softmax Pro 7 software. Antibody concentrations were compared with purified Ig standards. Before use in experiments, all reagents were titrated to ensure optimal performance.

### ELISPOT assay

Antibody-secreting cells were quantified using ELISPOT, following established methods^116,130^. Briefly, Multi-Screen HA Filtration plates were coated with unlabeled anti-isotype antibodies or whole killed influenza A/PR8/34 (200-400 HAU ml^−1^). After blocking and the addition of cell suspensions, antibody secretion was detected using biotinylated anti-isotype antibodies, followed by streptavidin–HRP. The resulting reaction was visualized using a previously described chemical method^130^. Plates were then enumerated with the AID EliSpot Reader System (Autoimmune Diagnostika).

### Immunofluorescence imaging and image analysis

For immunofluorescence analysis, B6.Cg-Tg(RP23-268L19-EGFP)2Mik/J (ChAT^BAC^eGFP, cat. no. 007902) mice were first perfused with 1× PBS until clear and then perfused with 4% paraformaldehyde. Tissues were subsequently post-fixed at 4 °C for an additional 2 h. After several washes in 1× PBS, the samples were immersed in 30% sucrose in 1× PBS overnight at 4 °C. The following day, the tissues were embedded in Tissue-Tek OCT (Sakura, cat. no. 4583) and frozen on dry ice. Frozen samples were sectioned at a thickness of 12 μm using a cryostat (Leica, cat. no. CM1850). Sections were initially washed in 1× PBS and then blocked in 10% goat serum (Invitrogen, cat. no. 01-6201) containing 0.5% Triton for 1 h. Subsequently, sections were incubated with primary antibodies overnight at 4 °C. The primary antibodies used were rat anti-B220 (clone RA3-6B2, 1:200, BioLegend, cat. no. 103202) and rabbit anti-F4/80 (clone D4C8V, 1:400, Cell Signaling Technology, cat. no. 30325T). The next day, slides were washed in 1× PBS 3× and then incubated with Alexa Fluor-conjugated, highly cross-adsorbed secondary antibodies along with DAPI (Sigma-Aldrich) for nuclear counterstaining. The goat-derived Alexa Fluor-conjugated secondary antibodies used were anti-rat 647 (1:1,000, Invitrogen, cat. no. A21247) and anti-rabbit 546 (1:1,000, Invitrogen, cat. no. A10010). Endogenous ChAT-GFP was also examined. Tissue sections were mounted with Aqua-Mount (epredia) and fluorescence images were acquired using a Zeiss 780 LSM confocal microscope. Images were acquired as tiled image stacks, covering 10- to 20-µm sections in an *x–y* plane. For further analysis, the images were analyzed using FIJI (ImageJ). For quantification, distances were normalized to a known reference and the measurement tool was used to calculate the distance between neighboring cells.

### Statistical analysis and reproducibility

Statistical analyses were conducted using either GraphPad Prism v.8-10 or DESeq2, as specified. Details about the statistical methods employed for each analysis are provided within the corresponding figure legends. The precise sample size (*n*) for each experiment, along with the number of times the experiment was performed and repeated to ensure reproducibility and statistical significance, are also outlined within the figure legends. In summary, most experiments were repeated at least twice and are presented as pooled data or as representatives of two or more repetitions. Blinding procedures were deemed unnecessary for the present study, because analyses were conducted using predefined criteria or computationally generated values, with the exception of lung tissue analysis (Fig. 5b), where blinding was implemented with a single-blinded approach by an expert veterinary pathologist.

### Reporting summary

Further information on research design is available in the Nature Portfolio Reporting Summary linked to this article.

## Online content

Any methods, additional references, Nature Portfolio reporting summaries, source data, extended data, supplementary information, acknowledgements, peer review information; details of author contributions and competing interests; and statements of data and code availability are available at 10.1038/s41590-025-02124-8.

## Supplementary information

Supplementary Information Supplementary Data 3 Immunofluorescence images.

Reporting Summary

Supplementary Data ScRNA-seq relative gene expression for cluster identification, differential gene expression and GSEA.

## Source data

Source Data Fig. 1 Statistical source data.

Source Data Fig. 2 Statistical source data.

Source Data Fig. 3 Statistical source data.

Source Data Fig. 4 Statistical source data.

Source Data Fig. 5 Statistical source data.

Source Data Fig. 6 Statistical source data.

Source Data Fig. 7 Statistical source data.

Source Data Extended Data Fig. 1 Statistical source data.

Source Data Extended Data Fig. 2 Statistical source data.

Source Data Extended Data Fig. 3 Statistical source data.

Source Data Extended Data Fig. 4 Statistical source data.

Source Data Extended Data Fig. 5 Statistical source data.

Source Data Extended Data Fig. 6 Statistical source data.

Source Data Extended Data Fig. 7 Statistical source data.

Source Data Extended Data Fig. 8 Statistical source data.

Source Data Extended Data Fig. 9 Statistical source data.

## Data Availability

All data are available within the paper and Supplementary Information. ScRNA-seq data have been submitted to the Gene Expression Omnibus (accession no. GSE290363). ScRNA-seq GSEA analysis gene sets were obtained from the Molecular Signatures Database. Source data are provided with this paper.
