# Supplementary material for: B cells modulate lung antiviral inflammatory responses via the neurotransmitter acetylcholine
Source: Nat Immunol. 2025 Apr 22;26(5):775–89. doi: 10.1038/s41590-025-02124-8 (PMC12043518; doi:10.1038/s41590-025-02124-8)
Supplement: Supplementary file 1 — Supplementary Data 3 Immunofluorescence images. [file 41590_2025_2124_MOESM1_ESM.pdf]

nature immunology

Supplementary information

https://doi.org/10.1038/s41590-025-02124-8

# B cells modulate lung antiviral inflammatory responses via the neurotransmitter acetylcholine

In the format provided by the authors and unedited

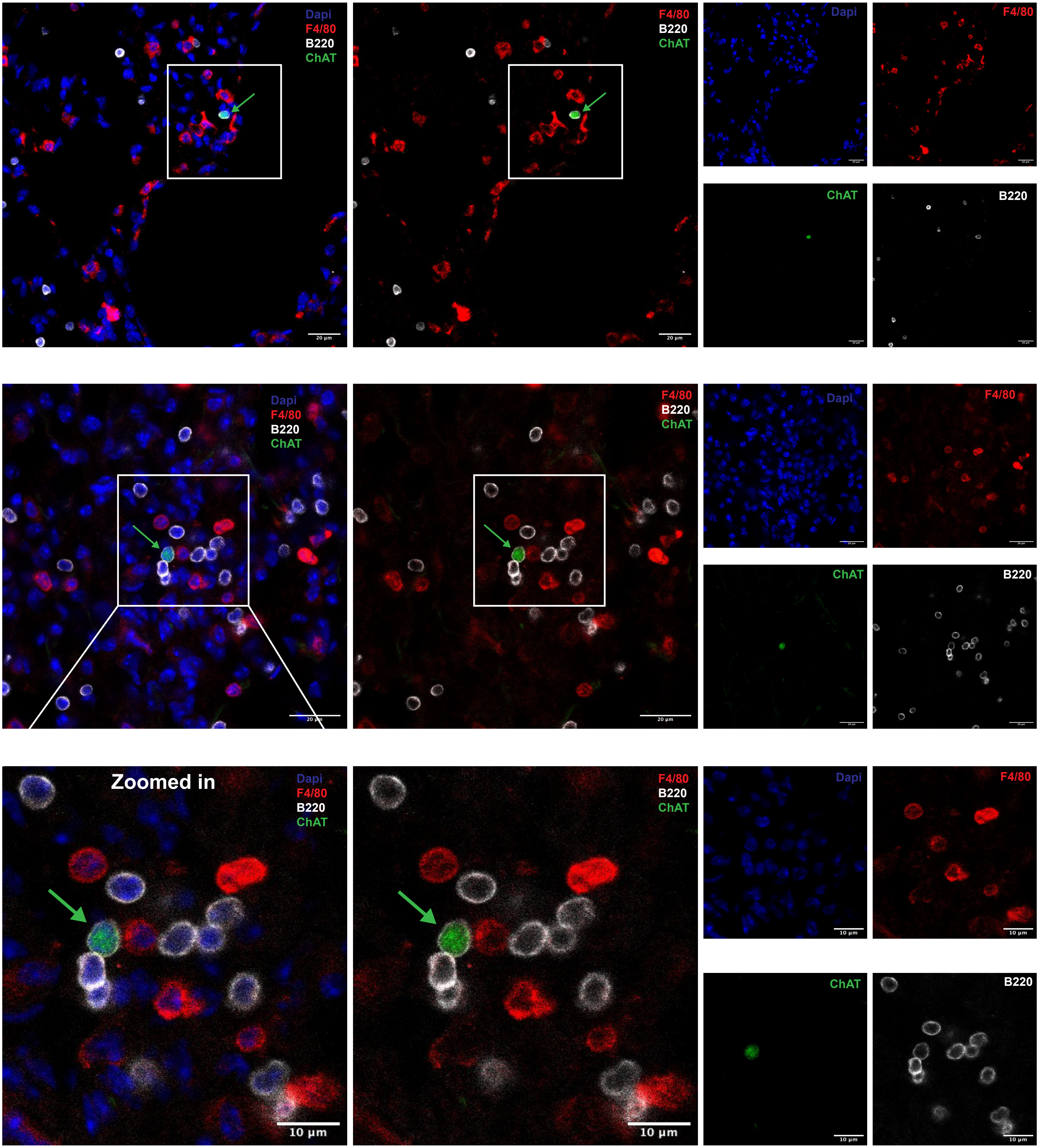

Dapi
F4/80
B220
ChAT
20 µm
F4/80
B220
ChAT
20 µm
Dapi
F4/80
ChAT
B220
Dapi
F4/80
B220
ChAT
20 µm
F4/80
B220
ChAT
20 µm
Dapi
F4/80
ChAT
B220
Zoomed in
Dapi
F4/80
B220
ChAT
10 µm
F4/80
B220
ChAT
10 µm
Dapi
F4/80
10 µm
10 µm
ChAT
B220
10 µm
10 µm

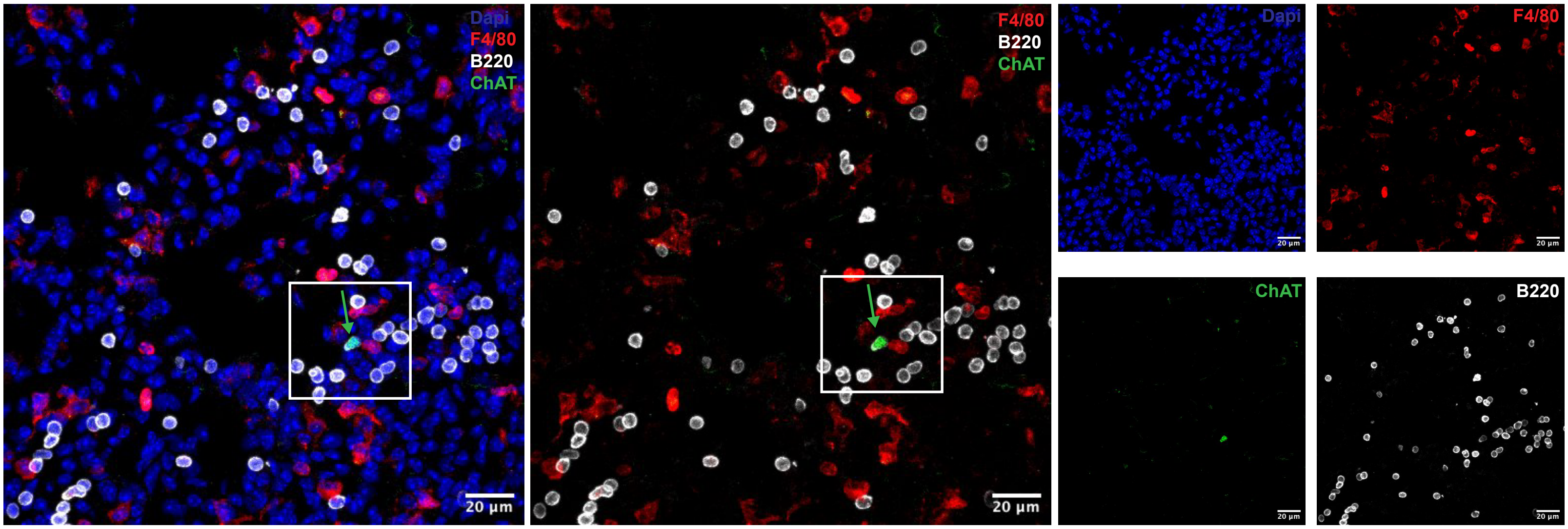

Dapi
F4/80
B220
ChAT
20 µm
F4/80
B220
ChAT
20 µm
Dapi
F4/80
ChAT
B220
