# Supplementary material for: B cells modulate lung antiviral inflammatory responses via the neurotransmitter acetylcholine
Source: Nat Immunol. 2025 Apr 22;26(5):775–89. doi: 10.1038/s41590-025-02124-8 (PMC12043518; doi:10.1038/s41590-025-02124-8)
Supplement: Supplementary file 2 — Reporting Summary [file 41590_2025_2124_MOESM2_ESM.pdf]

nature portfolio

Corresponding author(s): Nicole Baumgarth

Last updated by author(s): 2025/01/25

Reporting Summary

Nature Portfolio wishes to improve the reproducibility of the work that we publish. This form provides structure for consistency and transparency in reporting. For further information on Nature Portfolio policies, see our Editorial Policies and the Editorial Policy Checklist.

Statistics

For all statistical analyses, confirm that the following items are present in the figure legend, table legend, main text, or Methods section.

n/a	Confirmed	
☐	☒	The exact sample size (*n*) for each experimental group/condition, given as a discrete number and unit of measurement
☐	☒	A statement on whether measurements were taken from distinct samples or whether the same sample was measured repeatedly
☐	☒	The statistical test(s) used AND whether they are one- or two-sided<br>*Only common tests should be described solely by name; describe more complex techniques in the Methods section.*
☒	☐	A description of all covariates tested
☐	☒	A description of any assumptions or corrections, such as tests of normality and adjustment for multiple comparisons
☐	☒	A full description of the statistical parameters including central tendency (e.g. means) or other basic estimates (e.g. regression coefficient) AND variation (e.g. standard deviation) or associated estimates of uncertainty (e.g. confidence intervals)
☐	☒	For null hypothesis testing, the test statistic (e.g. *F*, *t*, *r*) with confidence intervals, effect sizes, degrees of freedom and *P* value noted<br>*Give P values as exact values whenever suitable.*
☒	☐	For Bayesian analysis, information on the choice of priors and Markov chain Monte Carlo settings
☒	☐	For hierarchical and complex designs, identification of the appropriate level for tests and full reporting of outcomes
☐	☒	Estimates of effect sizes (e.g. Cohen's *d*, Pearson's *r*), indicating how they were calculated

Our web collection on statistics for biologists contains articles on many of the points above.

Software and code

Policy information about availability of computer code

Data collection

Flow cytometry data was collected on: BD FACSymphony A3 and BD FACS Aria (sorting)
qRT-PCR Data was collected in Applied Biosystems QuantStudio 5/6 Flex
ELISPOT data was collected on AID EliSpot Reader System (Autoimmune Diagnostika)
ELISA data was collected using Molecular Devices SpectraMax M5 and quantified using Softmax Pro 7

Data analysis

Flow cytometry data was collected on BD FACSymphony A3 in FACS Diva v8.0.3 software and analyzed using FlowJo v9 and above

qRT-PCR data was quantified using QuantStudio Realtime PCR.

Immunoluorescence imaging was quantified using FIJI (ImageJ2 version 2.14.0/54f)

All other Statistical analyses were conducted using either GraphPad Prism v8-10 or DESeq2, as specified.

For single cell analysis, automatically called cells were further filtered to ensure usage of high-quality droplets with captured cells. RNA barcodes were filtered on Total UMI Count (> 500 UMIs), feature count (> 250 features), and percentage of mitochondrial genes (< 25%). Signac v1.9.0129 was used to determine nucleosome signaling and transcription start site enrichment scores. ATAC barcodes were filtered on number of fragments mapping to peak regions (> 3,000 and < 20,000), percentage of fragments mapping to peak regions (> 15%), nucleosome signal scores (< 4) and transcription start site scores (> 1).
Seurat v4.3.0130 and Signac v1.9.0129 used for handling of normalization, identification of variable genes, scaling, principal component analysis, UMAP dimensional reduction, and SNN generation followed by Leiden clustering for RNAseq and ATACseq data respectively. Batch effect correction was performed using Harmony. Clusters were identified using a combination of marker genes and differential expression

comparing each cluster to all other cells in the data set. Differential expression analyses were performed using Mann-Whitney U test. fgsea v1.24.0132 was used to run gene set enrichment analysis (GSEA). Features were ranked by -log(pvalue) * sign(foldchange).
Percentages of cells by cluster and sample are the percentage of cells in a given cluster compared to the total number of cells per sample. Statistical comparisons shown are from t-test comparing the percentages.
Intercellular signaling analyses are based Domino. UCell was used to generate transcription factor activation scores by cell using transcription factor target gene sets from the Molecular Signatures Database. ComplexHeatmap v2.14.0136 and circlize v0.4.15137 were used for visualizations.

For manuscripts utilizing custom algorithms or software that are central to the research but not yet described in published literature, software must be made available to editors and reviewers. We strongly encourage code deposition in a community repository (e.g. GitHub). See the Nature Portfolio guidelines for submitting code & software for further information.

Data

Policy information about availability of data

All manuscripts must include a data availability statement. This statement should provide the following information, where applicable:

- Accession codes, unique identifiers, or web links for publicly available datasets
- A description of any restrictions on data availability
- For clinical datasets or third party data, please ensure that the statement adheres to our policy

All data is available within the paper, supplemental information and sources. Single cell RNA-Seq GSEA analysis gene sets were obtained from the Molecular Signatures Database

Research involving human participants, their data, or biological material

Policy information about studies with human participants or human data. See also policy information about sex, gender (identity/presentation), and sexual orientation and race, ethnicity and racism.

Reporting on sex and gender	N/A
Reporting on race, ethnicity, or other socially relevant groupings	N/A
Population characteristics	N/A
Recruitment	N/A
Ethics oversight	N/A

Note that full information on the approval of the study protocol must also be provided in the manuscript.

Field-specific reporting

Please select the one below that is the best fit for your research. If you are not sure, read the appropriate sections before making your selection.

☒ Life sciences ☐ Behavioural & social sciences ☐ Ecological, evolutionary & environmental sciences

For a reference copy of the document with all sections, see nature.com/documents/nr-reporting-summary-flat.pdf

Life sciences study design

All studies must disclose on these points even when the disclosure is negative.

Sample size	The sample size was determined based on a pilot study (n=2–4), and the experiments were repeated to ensure data reproducibility.
Data exclusions	No data points were excluded, except for cases of influenza A infections where mice exhibited weight loss and sickness requiring euthanasia in accordance with ACUC protocols at the University of California, Davis, and Johns Hopkins University.
Replication	All experiments in this study were repeated at least twice and are presented either as pooled data or as representative results from two or more repetitions. The exact sample size (n) for each experiment, along with the number of repetitions performed to ensure reproducibility and statistical significance, is specified. The only exception is the single-cell RNA sequencing (scRNA-Seq) experiment, which was conducted once. The scRNA-Seq data was validated by qRT-PCR and further confirmed at the protein level. To enhance reproducibility and minimize mouse-to-mouse variation, cells for the scRNA-Seq experiment were pooled from two mice per group per time point.
Randomization	Randomization was not applicable to this study, as covariates (e.g., sex, age, treatment concentrations, cell numbers) were explicitly matched between experimental and control groups.
Blinding	Blinding procedures were deemed unnecessary for this study, as analyses were conducted using predefined criteria or computationally

Blinding | generated values, with the exception of lung tissue analysis (Fig. 5b), where blinding was implemented with a single-blinded approach by an expert veterinary pathologist.

Reporting for specific materials, systems and methods

We require information from authors about some types of materials, experimental systems and methods used in many studies. Here, indicate whether each material, system or method listed is relevant to your study. If you are not sure if a list item applies to your research, read the appropriate section before selecting a response.

Materials & experimental systems

n/a	Involved in the study
☐	☒ Antibodies
☒	☐ Eukaryotic cell lines
☒	☐ Palaeontology and archaeology
☐	☒ Animals and other organisms
☒	☐ Clinical data
☒	☐ Dual use research of concern
☒	☐ Plants

Methods

n/a	Involved in the study
☒	☐ ChIP-seq
☐	☒ Flow cytometry
☒	☐ MRI-based neuroimaging

Antibodies

Antibodies used

A supplemental list of antibodies and fluorescent dyes/antigen conjugations is given as a supplemental file.
All antibodies were tested and titrated prior to use to identify the best dilution that gave the highest differential fluorescence intensity between the negative and positive cell fraction using mouse spleen, bone marrow or peritoneal/pleural cavity washout cells, as appropriate. Dilutions varied between reagents and lots but typically ranged between 1:25 and 1:800, the latter especially if made in-house. Because the work was done over 6 years and with many different lots of the same reagents, individual dilutions are not provided.

Validation

All antibodies were purchased from companies well recognized and established for antibody manufacturing and scale. Antibodies were always tested and titrated prior use in experiments. Further validation of antibodies can be found from the product website using the reference number written in the supplemental file. Validation of flow cytometry staining was achieved using FMO controls and co-stains. For ELISPOT, anti-Ig antibodies were tested for specificity on purified murine antibodies of different isotypes and non-murine antibodies/serum for cross-adsorption by manufacturer.
List of antibody, clone and REF used in this study:
Antibody reagent Clone Source and REF
Anti-mouse CD3-AF700 17A2 BD Biosciences 561388
Anti-mouse CD4-PacBlue GK1.5 Made in-house
Anti-mouse CD4-BIOT GK1.5 Made in-house
Anti-mouse CD5-PE 53-7.3 Made in-house
Anti-mouse CD8a-PacBlue 53-6.7.3.1 Made in-house
Anti-mouse CD8a-BIOT 53-6.7.3.1 Made in-house
Anti-mouse CD9-BIOT MZ3 BioLegend 124803
Anti-mouse CD11b Biotin M1/70 Made in-house
Anti-mouse CD11b BV605 M1/70 BioLegend 101237
Anti-mouse CD11c-BUV737 N418 Invitrogen 3670-114-82
Anti-mouse CD19-PECF594 D3 BD Bioscience 562329
Anti-mouse CD19-BUV737 D3 BD Biosciences 612782
Anti-mouse CD19-BV786 D3 BD Biosciences 563333
Anti-mouse CD23 BV605 B3B4 BD Biosciences 747727
Anti-mouse CD24-BV711 M1/69 BD Biosciences 563450
Anti-mouse CD38-PE-DAZZLE594 90 BioLegend 102729
Anti-mouse CD43-APC S7 BD Biosciences 560663
Anti-mouse CD43-BV650 S7 BD Biosciences 740464
Anti-mouse CD44-BV711 IM7 BioLegend 103057
Anti-mouse CD45R-BV605 RA3-6B2 BD Biosciences 563708
Anti-mouse CD45R-BUV661 RA3-6B2 BD Biosciences 612972
Anti-mouse CD64-APC-eF780 X54-5/7.1 Invitrogen 47-0641-82
Anti-mouse CD62L-PE MEL-14 BioLegend 104407
Anti-mouse CD86-BV650 GL-1 BD Biosciences 564200
Anti-mouse CD90.2-BIOT 53-2.1 Made in-house
Anti-mouse CD90.2-PacBlue 53-2.1 Made in-house
Anti-mouse F4/80-BIOT BM-8 Made in-house
Anti-mouse F4/80-PacBlue BM-8 Made in-house
Anti-mouse F4/80-PECy5 BM-8 Biolegend 123111
Anti-mouse Ly6G/C-PacBlue RB6-8C5 or Gr-1 Made in-house
Anti-mouse Ly6G/C-BIOT RB6-8C5 or Gr-1 Made in-house
Anti-mouse Ly6G-BV421 1A8 BioLegend 127627
Anti-mouse NK1.1-PacBlue PK136 Made in-house
Anti-mouse NK1.1-BIOT PK136 Made in-house

Anti-mouse IgM-APC 331 Made in-house
Anti-mouse IgM-FITC 331 Made in-house
Anti-mouse IgM-APC-Cy7 331 Made in-house
Anti-mouse CD137 BV605 281-2 BD Biosciences 563147
Anti-mouse IRF4-PECy7 IRF4.3E4 Biolegend 646413
Anti-mouse IRF8-PerCP-eF710 V3GYWCH Invitrogen 46-9852-82
Anti-mouse CD95-BUV395 Jo2 BD Biosciences 740254
Anti-mouse TACI-PE 8F10 BioLegend 133403
Anti-mouse TACI-BV421 8F10 BD Biosciences 742840
Anti-mouse CD49b-BIOT DX5 BD Biosciences 553856
Anti-mouse Ly-51-PE BP-1 BioLegend 108307
Anti-mouse SiglecF-PerCP-eF710 1RNM44N Invitrogen 46-1702-82
Anti-mouse TNFa-FITC MP6-XT22 BD Biosciences 554418
Anti-mouse Ly6C-BV785 HK1.4 BioLegend 128041
Anti-mouse CD45.1-BUV395 A20 BD Biosciences 565212
Anti-mouse CD45.2-BUV661 104 BD Biosciences 741516

Animals and other research organisms

Policy information about studies involving animals; ARRIVE guidelines recommended for reporting animal research, and Sex and Gender in Research

Laboratory animals

Male and female 8-14 wk-old C57BL/6J (CD45.2 #000664), C57BL/6J-Ptprcem6Lutzy/J (JAXBoy CD45.1 #033076), B6.Cg-Tg(RP23-268L19-EGFP)2Mik/J (ChATBACeGFP #007902), B cell-deficient (uMT #002288), B6;129-Chattm1Jrs/J (ChATflox #016920), B6-C(Cg)-Cd79atm1(cre)Reth/EhobJ (Mb1-Cre on C57BL/6 #020505), B6.Cg-Tg(Lck-cre)548Jxm/J (Lck-Cre 548-O #003802) B6.129S4-Ccr2tm1Ifc/J (ccr2-/- #004999) mice were commercially obtained from The Jackson Laboratories.
Strains B6;129-Chattm1Jrs/J (ChATflox #016920), B6-C(Cg)-Cd79atm1(cre)Reth/EhobJ (Mb1-Cre on C57BL/6 #020505), B6.Cg-Tg(Lck-cre)548Jxm/J (Lck-Cre 548-O #003802) were initially provided by Drs. Colin Reardon and Kathrin Murray (UC Davis) and then continued to be bred in the animal facilities at Johns Hopkins University.
B6;129-Chattm1Jrs/J (ChATflox #016920) were bred with B6-C(Cg)-Cd79atm1(cre)Reth/EhobJ (Mb1-Cre on C57BL/6 #020505) to generate a B cell-specific deletion of chat and with B6.Cg-Tg(Lck-cre)548Jxm/J (Lck-Cre 548-O #003802) to generate a T cell-specific deletion of chat. C57BL/6J (CD45.2 #000664), C57BL/6J-Ptprcem6Lutzy/J (JAXBoy CD45.1 #033076) were bred to generate a CD45.1/2 strain for adoptive transfer experiments.
All mice were housed in SPF conditions in ventilated filtertop cages with food and water ad libitum at the University of California, Davis and the Johns Hopkins Bloomberg School of Public Health. Euthanasia was done by overexposing mice to CO2. All studies involving mice were conducted in compliance with, and after approval of protocols by the UC Davis Institutional Animal Care and Use Committee (IACUC) and by the Johns Hopkins University Animal Care and Use Committee (ACUC).
Male and female 8-14 wk-old C57BL/6J (CD45.2 #000664), C57BL/6J-Ptprcem6Lutzy/J (JAXBoy CD45.1 #033076), B6.Cg-Tg(RP23-268L19-EGFP)2Mik/J (ChATBACeGFP #007902), B cell-deficient (uMT #002288), B6;129-Chattm1Jrs/J (ChATflox #016920), B6-C(Cg)-Cd79atm1(cre)Reth/EhobJ (Mb1-Cre on C57BL/6 #020505), B6.Cg-Tg(Lck-cre)548Jxm/J (Lck-Cre 548-O #003802) B6.129S4-Ccr2tm1Ifc/J (ccr2-/- #004999) mice were commercially obtained from The Jackson Laboratories.
Strains B6;129-Chattm1Jrs/J (ChATflox #016920), B6-C(Cg)-Cd79atm1(cre)Reth/EhobJ (Mb1-Cre on C57BL/6 #020505), B6.Cg-Tg(Lck-cre)548Jxm/J (Lck-Cre 548-O #003802) were initially provided by Drs. Colin Reardon and Kathrin Murray (UC Davis) and then continued to be bred in the animal facilities at Johns Hopkins University.
B6;129-Chattm1Jrs/J (ChATflox #016920) were bred with B6-C(Cg)-Cd79atm1(cre)Reth/EhobJ (Mb1-Cre on C57BL/6 #020505) to generate a B cell-specific deletion of chat and with B6.Cg-Tg(Lck-cre)548Jxm/J (Lck-Cre 548-O #003802) to generate a T cell-specific deletion of chat. C57BL/6J (CD45.2 #000664), C57BL/6J-Ptprcem6Lutzy/J (JAXBoy CD45.1 #033076) were bred to generate a CD45.1/2 strain for adoptive transfer experiments.
All mice were housed in SPF conditions in ventilated filtertop cages with food and water ad libitum at the University of California, Davis and the Johns Hopkins Bloomberg School of Public Health. Mice were housed on a 6:30am/9pm ON/OFF light cycle, 68-76F degrees and 30%-70% humidity standards. Euthanasia was done by overexposing mice to CO2. All studies involving mice were conducted in compliance with, and after approval of protocols by the UC Davis Institutional Animal Care and Use Committee (IACUC) and by the Johns Hopkins University Animal Care and Use Committee (ACUC).

Wild animals

This study did not involve wild animals

Reporting on sex

Both male and female mice were randomly assigned to experimental groups as no overt differences were observed between sexes in our analysis, except for the known sexual dimorphism in IgM serum levels, for which male and female mice were analyzed separately (Extended Data Fig. 6b).

Field-collected samples

No field collected samples were used in this study

Ethics oversight

All studies involving mice were conducted in compliance with, and after approval of protocols by the UC Davis Institutional Animal Care and Use Committee (IACUC) and by the Johns Hopkins University Animal Care and Use Committee (ACUC).

Note that full information on the approval of the study protocol must also be provided in the manuscript.

Plants

Seed stocks	*Report on the source of all seed stocks or other plant material used. If applicable, state the seed stock centre and catalogue number. If plant specimens were collected from the field, describe the collection location, date and sampling procedures.*
Novel plant genotypes	*Describe the methods by which all novel plant genotypes were produced. This includes those generated by transgenic approaches, gene editing, chemical/radiation-based mutagenesis and hybridization. For transgenic lines, describe the transformation method, the number of independent lines analyzed and the generation upon which experiments were performed. For gene-edited lines, describe the editor used, the endogenous sequence targeted for editing, the targeting guide RNA sequence (if applicable) and how the editor was applied.*
Authentication	*Describe any authentication procedures for each seed stock used or novel genotype generated. Describe any experiments used to assess the effect of a mutation and, where applicable, how potential secondary effects (e.g. second site T-DNA insertions, mosiacism, off-target gene editing) were examined.*

Flow Cytometry

Plots

Confirm that:

☒ The axis labels state the marker and fluorochrome used (e.g. CD4-FITC).

☒ The axis scales are clearly visible. Include numbers along axes only for bottom left plot of group (a 'group' is an analysis of identical markers).

☒ All plots are contour plots with outliers or pseudocolor plots.

☒ A numerical value for number of cells or percentage (with statistics) is provided.

Methodology

Sample preparation	Lymph node and spleen cell suspensions were prepared as previously outlined (REF 128 in study). Briefly, tissues were ground between the frosted parts of two microscope slides and then incubated in ACK lysis buffer for 1 minute on ice to eliminate erythrocytes. Subsequently, the cells were passed through a 50 µm nylon filter and diluted for staining.<br>For lung tissue collection, lungs were harvested after left ventricle perfusion of the heart and then mechanically and chemically digested. The lungs were placed in 3 mL of DMEM F12 1X with 10% NCS in gentleMACS™ M Tubes (Milenty, # 130-093-236) and processed using a gentleMACS dissociator m_Lung_02 twice (Milteny). Following this, the lungs were incubated with DNAse I (50 U/mL) (Worthington-Biochem # LS002139) and Collagenase, Type I (250 U/mL) (Worthington-Biochem # LS004196) for 25 minutes at 37°C at 220 rpm shaking. After incubation, the lungs underwent another round of processing using the m_lung_02 program. The resulting cells were passed through a 50 µm nylon filter and diluted for staining, similar to lymph node and spleen preparations.
Instrument	BD FACSymphony A3 and BD FACS Aria (sorting)
Software	FACS Diva v8.0.3, FlowJo v9 or above
Cell population abundance	For sorting with FACS Aria, cells were reanalyzed after sorting, achieving and confirming a purity of >95%. For AUTOMACS sorting, a purity of over 90% was achieved unless otherwise specified in the study.
Gating strategy	For all flow cytometry gating strategies, leukocytes were first gated based on FSC-A and SSC-A, with doublets excluded via FSC-A and FSC-H. Live/dead exclusion was performed using fixable viability dye Aqua or IR780. Cell populations were defined as described in their respective figure legends. A gating strategy figure is provided as Extended Data Fig. 2.

☒ Tick this box to confirm that a figure exemplifying the gating strategy is provided in the Supplementary Information.
